# Multi-Stimuli-Responsive Tadpole-like Polymer/Lipid Janus Microrobots for Advanced Smart Material Applications

**DOI:** 10.1021/acsami.3c18826

**Published:** 2024-02-15

**Authors:** Burcu Okmen Altas, Cansu Goktas, Guliz Topcu, Nihal Aydogan

**Affiliations:** Department of Chemical Engineering, Hacettepe University, Beytepe, 06800 Ankara, Turkey

**Keywords:** Janus micromotor, anisotropic geometry, multistimuli-responsive, tadpole-like geometry, NIR light stimulation, magnetic field propulsion, Fenton reaction, degradation of methylene blue

## Abstract

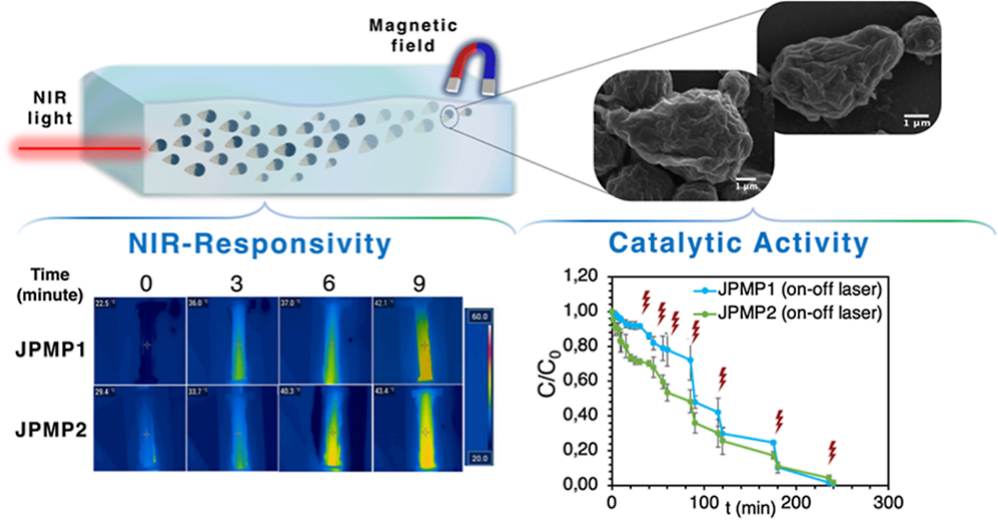

Microrobots are of significant interest due to their smart transport capabilities, especially for precisely targeted delivery in dynamic environments (blood, cell membranes, tumor interstitial matrixes, blood–brain barrier, mucosa, and other body fluids). To perform a more complex micromanipulation in biological applications, it is highly desirable for microrobots to be stimulated with multiple stimuli rather than a single stimulus. Herein, the biodegradable and biocompatible smart micromotors with a Janus architecture consisting of PrecirolATO 5 and polycaprolactone compartments inspired by the anisotropic geometry of tadpoles and sperms are newly designed. These bioinspired micromotors combine the advantageous properties of polypyrrole nanoparticles (NPs), a high near-infrared light-absorbing agent with high photothermal conversion efficiency, and magnetic NPs, which respond to the magnetic field and exhibit multistimulus-responsive behavior. By combining both fields, we achieved an “on/off” propulsion mechanism that can enable us to overcome complex tasks and limitations in liquid environments and overcome the limitations encountered by single actuation applications. Moreover, the magnetic particles offer other functions such as removing organic pollutants via the Fenton reaction. Janus-structured motors provide a broad perspective not only for biosensing, optical detection, and on-chip separation applications but also for environmental water treatment due to the catalytic activities of multistimulus-responsive micromotors.

## 1 Introduction

Synthetic micro-/nanomotors with autonomous functionalities are of great interest due to their unique motion behavior, especially for active targeted delivery in biomimetic and intelligent transportation.^1−4^ However, the effective actuation and manipulation of micro-/nanomotors is prevented by some limiting factors, such as the low Reynolds number regime where viscosity and Brownian motion occur.^5−7^ To overcome the limitations of swimming, considering that all living systems can adapt to complex and variable living environments with their unique characteristics, many studies have focused on bioinspired micro-/nanosystems, such as the swimming of fish, opening and closing of flowers, and crawling of snakes while designing multifunctional platforms that actively respond to dynamic environmental factors.^8−11^ In addition, the nature-inspired systems have been combined with fundamental concepts, including the Marangoni effect (such as pH, temperature, magnetic field, electric field, ultrasonic field, and light field), self-diffusiophoresis, self-electrophoresis, and bubble propulsion, to stimulate and actuate micrometer-sized systems in a liquid environment.^5,12,13^ Single-stimulated micromotors are not suitable for complex environments, where complex movements cannot be achieved. In fact, multiple-stimulus-stimulated micromotors have begun to attract more and more attention than single-stimulated ones^14^ for active targeting due to their capability of overcoming various biological barriers (blood, cell membrane, tumor interstitial matrix, blood–brain barrier, mucosa, and other body fluids).^12,13^

Up to now, two methods have been used to prepare stimuli-responsive micromotor systems in a single step, such as the top-down and bottom-up methods. For example, the electrospinning method, which is a top-down method, can be used to prepare stimuli-responsive polymer-based micromotors from the polymer solution. On the other hand, the emulsion solvent evaporation method can also be used to prepare stimuli-responsive polymer-based micromotor systems with various compositions. The emulsion solvent evaporation method can be applied with different techniques, such as a single emulsion (oil in water emulsion, o/w) and double emulsion (oil in water in oil emulsion, W/O/W).^13^ The electrospinning method of polymer-based systems is relatively expensive due to the high cost and low production rate. In contrast to the top-down method, bottom-up approaches first attempt to prepare microscale particles that can be assembled to form complex structures.

To date, different types of microrobots have been prepared using bottom-up approaches in many micro-/nanorobot studies to fabricate structures due to their environmental compatibility, biocompatibility, microscale flexibility, and large-scale manufacturability.^15,16^ For example, micro-/nanorobots are also widely used in environmental sensing and remediation. Environmental sensing expresses the changes in the action behavior of micro- and nanomotors with certain pollutants. Orozco et al. prepared microrobots that can test the water quality. When water contains pollutants, the propulsion behavior of biocatalytic robots changes according to the test.^17^ Another example is the superhydrophobic alkanethiol-coated microrobots developed by Guix et al. The microrobots can collect oils in water and for other water/oil separation processes.^18^ Ji et al. proposed an active nanocatalyst model using magnetic nanomotor ensembles to enhance catalytic performance by the combined effects of the magnetic field and near-infrared (NIR) laser irradiation. As a result of their studies, they proved that the structures showed high catalytic activity under hybrid stimuli of the magnetic field and light to degrade pollutants in an aqueous solution.^19^ Foam-like structures Fe_3_O_4_/f-C_3_N_4_ were also prepared by Feng et al. A study was carried out for the cost-effective reduction of antibiotics in industrial wastewater treatment by increasing the catalytic activity of the structures by the magnetic field and light.^20^ Feng et al. also reported covalent organic framework-based micro-/nanomotors in another study. These structures were shown to be more efficient in the photocatalytic degradation of tetracycline compared to the traditional magnetic stirring method under visible light irradiation.^21^

In addition to environmental applications, Yang et al. prepared fishlike microrobots that respond to multiple stimulants. They demonstrated that the controllability and flexibility of their multistimulus-responsive bionic fish microrobots could enable them to overcome complex tasks and limitations in liquid environments.^1^ Similarly, Ahmed et al. designed microsized superparamagnetic particles inspired by neutrophils. They combined magnetic and acoustic propulsion to perform complex microcargo operations with a rolling motion along the walls of rectangular and circular microchannels.^10^ Although great progress has been made in micro-/nanomotor systems, many studies have demonstrated limited active delivery abilities, such as lack of multiple cargo delivery, uncontrolled cargo release, and nontarget cargo delivery due to their traditional symmetrical structure with a single structure. Therefore, it is of utmost importance to prepare them in terms of material compositions, sizes, and shapes.^14^

Furthermore, micro-/nano-delivery systems mostly have simple geometries such as spherical geometry (aspect ratio is close to 1.0) from the inorganic or organic materials.^22,23^ Still, recent studies have underlined that asymmetric carriers will gain more importance in the coming years. For example, Wang et al. showed that rodlike particles with an aspect ratio of 1.9 exhibit greater diffusion in mucus compared to their spherical counterparts with an aspect ratio of 1.0. Thus, they emphasized that the shape of particles plays an important role in their diffusion behavior in the biological porous media.^24^ However, depending on the application, it is necessary to consider the possible interaction of the particles with biological media and even the possibility of embolism.

From here, unlike the traditional delivery systems, Janus particles (JP) have attracted great interest in recent years in pioneering studies as biomedical robots due to their anisotropic geometry with unique compartments whose surfaces have two or more different physical properties within a single structure.^25−27^ Over the past few decades, micro-/nano-Janus motors have evolved with significant improvements in manufacturing and driving, and external fields can propel them. Particularly, besides the chemical composition of JP, their precisely tunable shape is one of the most important design parameters to mimic nature as it will significantly affect their motion speed and even their directions in fluid environments.^28,29^ The proportions of compartments and other processing conditions can be adjusted to change the shape of JPs from spherical to the anisotropic form.^27,30^ Because of their intrinsic immiscibility, polymers and lipids are promising candidates for phase separation in the formation of JPs.^31^ Despite the significant research efforts devoted to stimuli-responsive Janus micro-/nanomachines, multiresponsive, bioinspired, and organic material-based (polymer and lipid materials) JP that can be triggered by temperature and the magnetic field have never been studied.^32−34^ Herein, this study aimed to construct biomimetic robots, which were prepared by a bottom-up approach that can simulate similar motions in biological microswimmers such as tadpoles, sperm, bacteria, and algae to enhance diffusivity through barriers and biological hydrogels due to their asymmetric geometry (aspect ratio >1). For this purpose, Janus micromotors (JMs) based on polycaprolactone (PCL) as a polymer and Precirol ATO 5 as a lipid were newly designed to be propelled by magnetic/light movement by combining polypyrrole nanoparticles (PPNP) and magnetic NPs for cargo transport, which is the main novelty of this work. Precirol ATO 5, an FDA-approved lipid, is widely used in the preparation of solid or nanostructured lipid NPs for drug delivery purposes. Precirol ATO 5, which is biodegradable and biocompatible, is a mixture of monoglycerides, diglycerides, and triglycerides.^31^ PCL is also a biodegradable and biocompatible material, an FDA-approved polymer, used for various healthcare applications.^35^

There has been no investigation of organic-based, biocompatible (polymer and lipid materials) JP that are multistimulus-responsive and can be triggered by temperature and the magnetic field, despite significant advances in the literature with stimulus-responsive Janus micro-/nanomotors. This study reports on the unique design of JMs that exhibit multistimuli-responsive motion behavior via an anisotropic, polymer/lipid multicompartment structure for large-scale synthesis. External sources such as NIR light and magnetic field-driven JMs can move rapidly in the aqueous medium, and their “on/off” propulsion mechanism can be easily controlled with the magnetic field and NIR irradiation. In addition, JMs can provide other functions, such as the removal of organic pollutants via the Fenton reaction, the activity of which can be tuned to the multifunctional nature of the designed Janus structures. More importantly, compared with previously reported microrobot systems, the asymmetric JMs with magnetic propulsion and (NIR) light stimulation function, bearing compartments in their structure capable of the simultaneous and sequential release of loaded cargo, can not only be used as a widely adaptable platform to help advance the research and treatment of fatal diseases via targeted delivery but also have a broad perspective in the environmental application due to their catalytic activity. This shuttle, like microrobots, can also be used in various fields such as optical sensing, on-chip separation, detoxification, separation, cargo transport, electrochemical sensing, and biosensing applications.^36^

## 2 Experimental Section

### 2.1 Materials

Iron(III) chloride, iron(II) sulfate heptahydrate, sodium dodecyl benzenesulfonate (SDBS), 3,6-bis(diethylamino)-9-(2-octadecyloxycarbonyl) phenyl chloride (Rhodamine B), PCL (molecular weight 40,000–80,000 Da), dichloromethane (DCM), pyrrole monomer, tween 80, and 5,5-dimethyl-1-pyrroline-*N*-oxide (DMPO) were obtained from Sigma-Aldrich (Sigma, St. Louis, MO, USA) and were used unpurified. Geleol mono diglyceride and Precirol ATO 5 glycerol distearate type I EP were gifted by Gattefossé SAS (Saint-Priest Cedex, France) and used as received. Oleic acid was purchased from Fisher Chemicals. Ultrapure (UP) water with a resistivity of 18.2 MΩ·cm (Millipore Sigma, Burlington, MA, USA) was used in all experiments.

### 2.2 Design and Fabrication of JM Particles

Polymer/lipid JM particles were prepared using the double emulsion solvent evaporation method.^25−27^ The internal aqueous phase consisted of PPYNP dispersed in 0.5 mL of UP water. PPYNP-to-lipid mass ratios studied were ×0.5, ×1, and ×1.5 (w/w 0.06 for ×0.5). The oil phase was also prepared with a 1:1 mass ratio of PCL and Precirol ATO 5 into 7.5 mL of DCM with different amounts of magnetic nanoparticles (MNP). The MNP-to-lipid mass ratios studied were ×1, ×2, and ×3 (w/w 0.078 for ×1). To prepare the external phase, PVA and SDS or SDBS were dissolved at a mass ratio of 3:1, and the surfactant type was investigated (Table S1). First, the W/O emulsion was homogenized by using manual shaking for 4.5 min (see Table S1). Then, the double W/O/W emulsion was produced by adding the W/O emulsion to the external aqueous phase and emulsifying it using the same stirring technique as for the first emulsion. The resulting solution was magnetically stirred at a constant speed for 4 h to remove DCM. The final suspension was centrifuged and washed three times at 10,000 rpm for 5 min in a microcentrifuge MPW-55 and then washed with UP water to remove residual surfactants. The samples were kept at +4 °C for further analysis. The fabrication procedure of JM particles is demonstrated in Figure S1.

### 2.3 Synthesis of Polypyrrole Nanoparticles

First, PVA and FeCl_3_ were dissolved in 50 mL of UP water to prepare PPYNP as a photothermal agent.^37^ A transparent yellow solution was obtained. The system was allowed to reach equilibrium for 1 h, and the required amount of pyrrole was added to the solution. The solution turned black within a few minutes. It was stirred in an ice bath for 4 h. After polymerization, the system was warmed to room temperature. The product was separated by centrifugation and washed 3 times to remove impurities. Finally, the product obtained was dried at room temperature.^38^

### 2.4 Synthesis of Oleic Acid-Coated Magnetic Nanoparticles

FeCl_3_·6H_2_O and FeSO_4_ were dissolved in UP water to obtain a transparent yellow solution. Then, NaOH solution was added dropwise until pH = 10 was reached. Next, 100 μL of oleic acid was added to the solution and stirred for 1 h. To impart magnetic properties to the iron hydroxides, the solution was heated to 95 °C. HNO_3_ was added to the solution, which comes to room temperature naturally, and it was ensured that the pH was 5. The solution was washed four times with UP water and then with acetone to remove impurities (see Figure S2).^39^

### 2.5 Characterizations

The optical and fluorescence microscopy images were taken using an inverted microscope (Leica DMI 4000B). The images were captured using immersion oil with a 100× oil objective to achieve a higher magnification ratio. Samples were analyzed in suspension and dried at room temperature. Scanning electron microscopy (SEM, Bruker, Zeiss Evo 50) was used to observe the size and geometry of the JMPs. The samples were dried at room temperature by placing a drop on a clean aluminum stub. After gold deposition, the samples were then analyzed. Dynamic light scattering (DLS, ALV-CGS-3 Compact Goniometer) at an angle of 90° was used to determine the hydrodynamic radius and size distribution of MNP and PPYNP. At least six different batches were used for each DLS measurement. PPYNP was identified by Fourier transform infrared spectroscopy (Nicolet 6700 FTIR, Thermo Scientific). Surface tensions were determined at 20 °C by the pendant drop method using a Kruss Drop Shape Analyzer (DSA30, KRÜSS, Hamburg, Germany).

### 2.6 Trajectory and Velocity Measurements of JMs

JMs were dispersed in an aqueous solution and observed under a light microscope for the micromotor study. Trajectory and velocity measurements were performed using tracker video analysis. The NIR light-actuated motion was achieved by applying an 808 nm laser (CNI Model with PSU-III-LED, MDL-III-808–2.5 W), which was positioned near the micromotor solution at a distance of 10 cm. Additionally, the photothermal stability of the JMs was also examined. All micromotors were exposed to NIR light at a wavelength of 808 nm and a power of 2.5 W for 10 min. Subsequently, they were cooled to room temperature for two cycles.

### 2.7 Catalytic Activity of JMs

The JMs were dispersed in 2 mL of methylene blue (MB) solution, which had an initial concentration of 10 mg/L. The pH of the MB solution was adjusted to 2.5. MB degradation was initiated by the rapid addition of H_2_O_2_ (0.0017 v/v). The reaction mixture was kept in the absence of light at different temperatures (40 and 50 °C) during the experiment. At selected time intervals, aliquots (3.4 μL) were withdrawn, the MB absorbance was determined from a NanoDrop Spectrophotometer (NanoDropTM2000 Spectrophotometers, Thermo-Fisher Scientific, US), and the concentration values were calculated from the calibration curve (see Figure S3). The control experiments with the JMs without H_2_O_2_ or H_2_O_2_ without JMs were performed. To detect and scavenge ^•^OH produced by the Fenton reaction, methanol was used as reported in the literature.^40^ The free radical quenching tests were carried out by adding methanol (50 and 100 mM) to the solution, and the MB reduction efficacy of JPMPs was investigated as explained.

### 2.8 Electron Paramagnetic Resonance Analysis

Trapping experiments were performed to examine the contribution of the reactive oxygen species (ROS) generated by JPMP1 and JPMP2 to MB degradation.^20^ Electron paramagnetic resonance (EPR) measurements were conducted at room temperature using a Bruker EMX 131 X-Band ESR Spectrometer (Germany) operating at a frequency of 9.853 GHz and a microwave power of 10.0 mW. For EPR measurements, JPMP1 and JPMP2 (5.5 mg/mL) were dispersed in UP water (1.5 mL), including 20 mM H_2_O_2_. Then, the DMPO, which was used as a radical trapper, stock solution was added into the medium to have a final concentration of 125 mM. The resulting solution was transferred into a capillary of 1 mm diameter, which was then inserted in a quartz tube of 3 mm diameter. The quartz tube containing the capillary was placed in the cavity of the ESR spectrometer to determine the ROS. This time was referred to as zero time. ESR spectra were recorded just following the preparation of the solution and after 20 min.

### 2.9 Cytotoxicity of JPMPs

The MTT colorimetric assay was used to evaluate the cytotoxicity of JPMPs against L-929, a mouse fibroblast cell line. After 24 h of incubation, JPMPs were dispersed in the medium at concentrations of 5, 50, 100, 200, and 500 μg/mL. The cells were exposed to the particles for 24 h. Then, the suspensions were discarded, and the wells were washed twice with phosphate-buffered saline solution. The medium and MTT solution were added to each well, and the mixture was incubated for 2 h. The toxicity of the formulation was evaluated by using the values obtained for untreated cells as a reference and calculating the percentage of L-929 cell survival after treatment.

### 2.10 Statistical Analysis

Data are presented as mean ± standard deviation (SD) and all results were obtained from at least three replicates. One-way analysis of variance (ANOVA) followed by Tukey’s post hoc test for multiple comparisons was used to evaluate the dispersion of the data.

## 3 Results and Discussion

### 3.1 Preparation and Characterization of Polymer/Lipid JMs

In the present study, inspired by the tadpole and sperm geometry, smart JMs with a multistimulus-responsive motion behavior, which were based on combined magnetic and NIR light (808 nm) stimulation via anisotropic geometry, were designed to enhance diffusivity through cellular barriers and to overcome various biological obstacles.

The JMs were prepared by emulsion-solvent evaporation, and microstructures were obtained after solvent removal. The emulsion-solvent evaporation process represents a straightforward one-pot technique that is well-suited for the preparation of significant quantities of JMs from various polymers and other biodegradable materials. Moreover, this technique is easily scalable and requires minimal processing equipment.^27,41,42^ The design parameters can be easily modified by using this method. Because the functions of micromotors depend on their geometry, controlling their shape is critical for many potential applications, and it can be controlled by adjusting the interfacial tensions between different phases.^30,43^ For this purpose, the effect of the surfactant type in the external phase on the geometry of the JPs was systematically analyzed. Then, certain amounts of MNPs (Figure S1), which are hydrophobic in nature, were incorporated into the JPs to impart a magnetic propulsion capability to the prepared JPs. After that, PPYNP, which is a high NIR light-absorbing agent with high photothermal conversion efficiency under NIR light irradiation (808 nm),^44^ was used in particular amounts to add light responsiveness to the JMs.^37^ Finally, we combined the NIR stimulation and magnetic propulsion properties of polymer-/lipid-based JMs to perform complex targeted microparticle delivery and facilitate their diffusion in different biological environments. The JMs can achieve various programmable motions under the combined action of a magnetic field and NIR stimulation, paving the way for various applications. The physicochemical properties and composition of the JMs are shown in Table S1. Here, we used a low shear rate (manual shaking) during the emulsification process to prepare JMs to determine if it is possible to control the geometry and compartmentalization by changing the type of surfactant and easily characterized by using optical microscopy and SEM.

#### 3.1.1 Control of Geometry by Changing Surfactant Type in the External Phase

**Figure 1:**
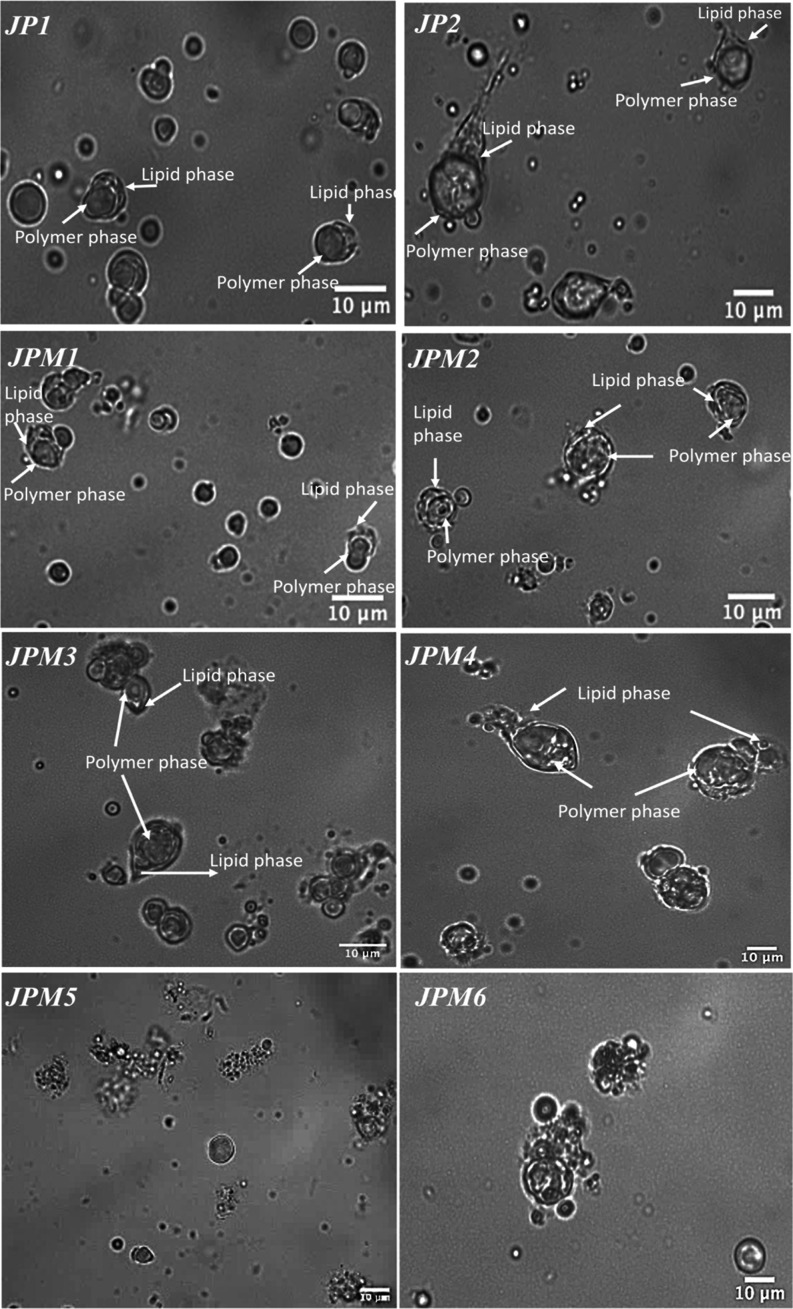
Light microscope images of JPs were prepared using different cosurfactants and different amounts of MNPs. JP1 and JP2 were prepared with PVA/SDBS and SDS/SDBS cosurfactants. JPM1, JPM2, JPM3, JPM4, JPM5, and JPM6 were prepared with different amounts of MNPs and cosurfactants. JPM1 and JPM2: external phase containing PVA/SDBS and SDS/SDBS cosurfactants, respectively (×1 MNPs), JPM3 and JPM4: external phase containing PVA/SDBS and SDS/SDBS cosurfactants, respectively (×2 MNPs), JPM5 and JPM6: external phase containing PVA/SDBS and SDS/SDBS cosurfactants, respectively (x3MNPs).

It has been emphasized in the literature that the morphology of the JPs can be predicted by determining interfacial tensions between the aqueous and the organic phases, which can be identified with the spreading coefficient theory (see eq 1 in the Supporting Information).^30^ The spreading coefficient can be controlled by adjusting the mass fraction and molecular weight of the organic compartments as well as the surfactant concentration and type.^42^ From here, the effect of the type of surfactant in the external phase on the geometry of the JP was analyzed using different stabilizers. PVA was chosen as a nonionic stabilizer, while SDBS and SDS were chosen as anionic surfactants.^45^ The surface tension values of the external phases are given in Figure S4. The surface tension of the external phase consisting of PVA and SDBS cosurfactants was determined as 24.7 ± 0.3 mN/m, while the surface tension of the external phase consisting of SDS and SDBS cosurfactants was determined to be 30.3 ± 0.2 mN/m. JP formulations were then prepared by combining PVA and SDS separately with SDBS to compare the effect of the geometry of PVA with that of SDS on the geometry. The results showed that the combination of PVA with SDBS (JP1) and the combination of SDS with SDBS (JP2) exhibited different JP geometries when they were used to prepare the external phase with UP water (see Figure 1). Since the use of SDS increased the interfacial tension between the organic phase and the water phase more than the use of PVA, it was considered that the tadpole-like structure was more clearly observed in JP2 (see Figure 1).^31^ Considering that the result is compatible with the literature, it is predicted that the observed interfacial areas in the geometries of the JPs shown in Figure 1 can also explain the surface tension values.^31^

When the PVA was used as the stabilizing agent, the droplets with the size of 14.68 ± 2.04 μm were initially formed. At the end of the solvent evaporation process, the droplet size decreased, and finally, particles with a diameter of 6.59 ± 1.80 μm and a length of 7.89 ± 2.53 μm were obtained. The aspect ratio of JP1 was calculated as ∼1.2 ± 0.3, which means that it has an asymmetric geometry (see Figure S5). On the other hand, when we used SDS as a stabilizing agent to form JPs, the droplet size was measured to be 12.60 ± 1.96 μm. The diameter of JP2 was determined as 8.49 ± 1.96 μm, and the length was analyzed as 14.19 ± 4.41 μm. Accordingly, JP2 has long tadpole-like tails with an aspect ratio of ∼1.7 ± 0.4 when the same mass fraction of SDS was used instead of PVA in the external phase (see Figure S5).

### 3.2 Formation of MNP-Loaded JMs

Considering that the magnetic field plays an essential role in providing motion to micromotor systems due to its noninvasive remote propulsion capability and favorable orientation capability,^46^ different amounts of MNPs (80.74 ± 7.90 nm), MNP-to-lipid mass ratios were studied ×1, ×2, and ×3 (w/w 0.078 for ×1, see Figures S6 and S7) were loaded on JP1 and JP2 which were prepared using different surfactants (see Table S1 and Figure 1). The diameter and length of JPM1 prepared by loading ×1 MNP were measured as 5.73 ± 1.16 and 6.60 ± 1.67 μm, respectively, after the DCM was removed from the solution (see Figure S7). On the other hand, the diameter of JPM2 was determined as 6.82 ± 2.15 μm, and the length was measured as 10.13 ± 3.05 μm (see Figure S7). Despite the addition of MNP to JPs, it is seen that both particles retain their asymmetric geometry, and JPM2 still has the highest asymmetry with an ∼1.67 ± 0.17 aspect ratio. The aspect ratio was also calculated as ∼1.19 ± 0.08 for JPM1. However, no movement was observed in JMs when a certain magnetic field was applied to JPM1 and JPM2 containing ×1MNP.

Thereafter, the experiments were repeated by doubling the amount of MNP (×2) added to the JPs relative to the amount of lipid. The diameter and length of JPM3 were analyzed as 5.76 ± 1.15 and 7.71 ± 2.21 μm, respectively, while the diameter and length of JPM4 were analyzed as 7.18 ± 1.81 and 8.75 ± 2.06 μm, respectively. From here, the aspect ratio values of the particles were determined as ∼1.22 ± 0.1 and ∼1.33 ± 0.02 for JPM3 and JPM4, respectively (see Figure S7). Although the diameter values of JPM3 and JPM4 remained almost the same, the aspect ratios decreased with doubling the amount of MNP (×2). Besides that, it was observed that when a certain magnetic field (480 mT) is applied to JPM3 and JPM4 containing ×2 MNP, these micromotors can move with the effect of the magnetic field.

**Figure 2:**
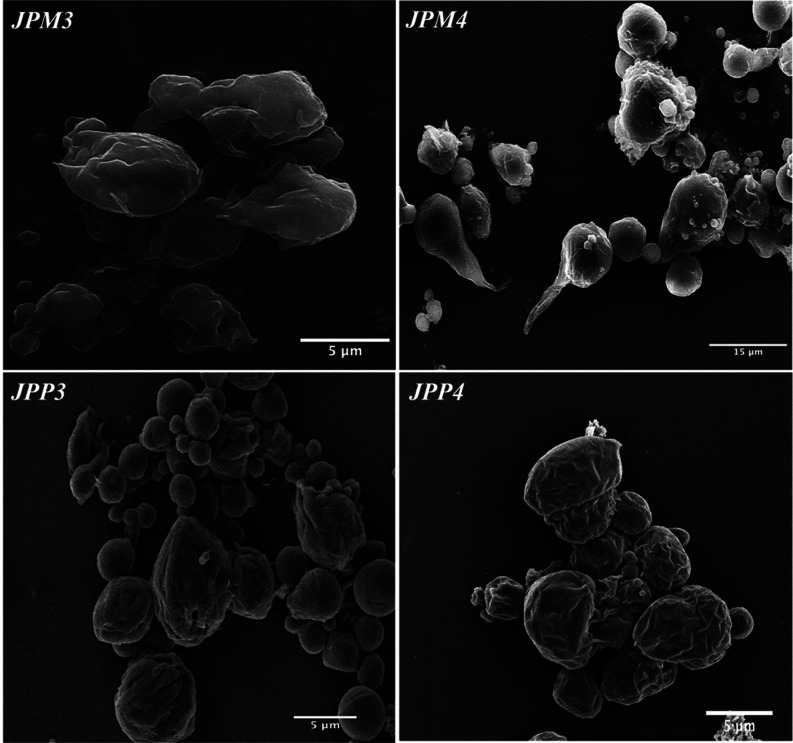
SEM images of single-stimulated JMs (JPM3, JPM4, JPP3, and JPP4). JPM3 and JPM4: external phase containing PVA/SDBS and SDS/SDBS cosurfactants, respectively (×2 MNPs), JPP3 and JPP4: external phase containing PVA/SDBS and SDS/SDBS cosurfactants, respectively (×1 PPYNP).

Finally, it was thought that increasing the amount of MNP added to the particles would increase the speed of the JPs, and the JPs were prepared again by tripling the amount of MNP (×3). Although droplet formation was observed for JPM5 and JPM6, it was observed that the particles could not maintain their structure and deteriorated after solvent evaporation (Figure 1). Therefore, the MNP-to-lipid mass ratio of ×2 was the upper limit for the preparation of MNP-loaded JMs. Based on these results, although the amount of MNP added to the particles changed, the droplet sizes and diameters remained almost the same for all analyzed particles. Considering the anisotropic structures (aspect ratios) and magnetic responsivity of the particles, we employed SEM for a more comprehensive understanding of the structural characteristics of JPM3 and JPM4. For this purpose, JPM3 and JPM4 were comparatively analyzed with polymer particles prepared with PCL without Precirol ATO 5 (see Figure 2 for JPM3 and JPM4 SEM images; also, see Figure S9 for PCL particles). Observing the images revealed a distinct contrast: while the surface of polymer particles formed solely from PCL presented a smooth and spherical shape, the surfaces of JPM3 and JPM4 loaded with ×2 MNP, which consisted of both PCL and Precirol ATO 5 compartments, exhibited a markedly uneven structure and an asymmetrical geometry. Similar to the data obtained from optical microscope images, the aspect ratios of JPM3 and JPM4 were measured as ∼1.18 ± 0.19 and ∼1.38 ± 0.25, respectively. The distributions of the diameter and length of JPM3 and JPM4 analyzed via SEM are given in Figure S8.

### 3.3 Formation of PPYNP-Loaded JMs

When a NIR light irradiates the microrobot, the JMs on its surface absorb the light energy and convert it into heat via the NIR light-absorbing agent, and the temperature gradient causes the microrobot to move. This is defined as “Thermophoresis, also known as thermodiffusion or the Ludwig-Soret effect”, which occurs in solutions that cause the microrobot to move through the temperature gradient.^47^ Considering that laser irradiation can trigger the photothermal effect of the light-responsive micromotors, leading to the propulsion of the whole structure,^37,48^ different amounts of PPYNP (50.86 ± 23 nm, Figure S10), which has a photothermal effect,^44^ were added to the JP1 and JP2 (see Table S1) to obtain JPPs (PPYNP-loaded JPs). PPYNP has received great attention in various fields and applications due to its high biocompatibility, photostability, and conductivity.^44^

**Figure 3:**
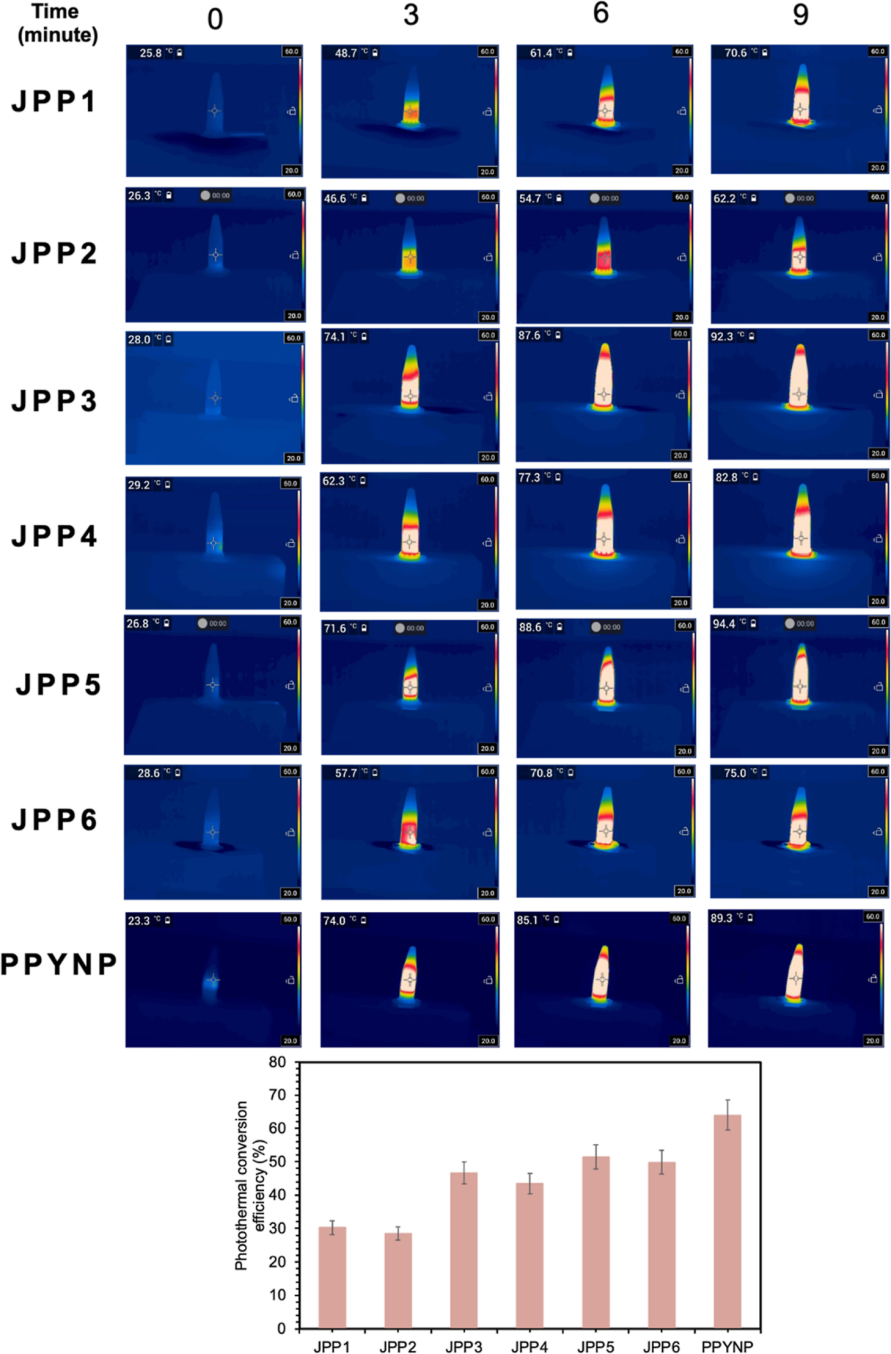
Thermal camera images and photothermal conversion efficiency values (%) of JMs containing different concentrations of PPYNP at 808 nm NIR irradiation and 2 W power. JPP1 and JPP2: external phase containing PVA/SDBS and SDS/SDBS cosurfactants, respectively (×0.5 PPYNP), JPP3 and JPP4: external phase containing PVA/SDBS and SDS/SDBS cosurfactants, respectively (×1 PPYNP), JPP5 and JPP6: external phase containing PVA/SDBS and SDS/SDBS cosurfactants, respectively (×1.5 PPYNP).

The light microscope images of the resulting structure are given in Figure S11. The droplet sizes of JPP1 and JPP2 containing 0.5 × 0.5 PPYNP were measured as 13.76 ± 3.35 and 12.93 ± 1.95 μm, respectively. After the solvent was removed, the average diameter and length for JPP1 and JPP′ were determined as 7.20 ± 1.69, 5.90 ± 1.33 μm, and 9.43 ± 2.49, 9.57 ± 2.07 μm, respectively (see Figure S12, the size distribution histogram). On the other hand, the photothermal conversion efficiency (η) values were calculated to be almost 30 ± 2.1 and 28 ± 1.9% for JPP1 and JPP2, respectively (see Figure 3 and eq 2 in the Supporting Information). This difference might be due to differences in the position of the compartments and the surface properties of the prepared micromotors.

In our study, the temperature increase of JP1, JP2, JPM3, and JPM4 upon NIR laser irradiation was determined to be 6–8 °C even after 9 min of irradiation, and no net movement was observed (see Figure S13). When the NIR light was also applied to the PPYNP-loaded JPs, the temperature increased, and movement of the micromotors was observed. In the literature, Zhang et al. evaluated that the light response of micromotors was reported to be related to the photothermal effect of the PPYNP.^37^ In another study, Zha et al. investigated the photothermal effect resulting from NIR laser irradiation (808 nm, 2 W) in the presence of varying concentrations of PPYNP in 3 mL of the solution for 10 min. They emphasized that the temperature elevation increased with the increasing concentration of PPYNP.^49^

To prove the different potentials of PPYNP, which were used because of their photothermal conversion ability to prepare JMs, both the temperature elevations and the photothermal conversion efficiency (η) values of JMs containing various amounts of PPYNP under 808 nm NIR irradiation were investigated. As the amount of PPYNP added to the JPs per lipid was doubled (×1), particles (JPP3 and JPP4) were prepared (see Table S1). The diameter and length for JPP3 were determined as 8.17 ± 3.19 and 9.50 ± 2.24 μm, respectively. On the other hand, the average diameter and length for JPP4 were also measured as 5.93 ± 0.87 and 9.08 ± 1.02 μm, respectively (see Figure S12 for the size distribution histogram). The photothermal conversion efficiency values were calculated as 47 ± 3.2 and 43 ± 3.0% for JPP3 and JPP4, respectively, as shown in Figure 3. Although no notable difference was observed in the size of the JMs, it was clearly seen that the photothermal conversion efficiency values increased with increasing PPYNP amount.^50^

To investigate whether there is a further increase in the photothermal conversion efficiency, higher amounts of PPYNP (1.5×) were used during the preparation of JPP5 and JPP6 (see Table S1). The diameter and length of JPP5 and JPP6 were analyzed as 7.16 ± 1.94 and 5.67 ± 0.90 and 9.12 ± 2.14 μm and 8.80 ± 2.07 μm, respectively (see Figure S12). The photothermal conversion efficiencies were determined as nearly 51 ± 3.6 and 49 ± 3.4% for JPP5 and JPP6, respectively, showing an elevated performance as expected. Considering the experimental data, it was found that JPP5 and JPP6 containing ×1.5 PPYNP had a similar photothermal conversion efficiency as that of JPP3 and JPP4 holding ×1.0 PPYNP.

Despite the increase in the amount of PPYNP contained in the JMs, an increase in the photothermal conversion efficiency and temperature difference of the JMs was not observed, as expected. This may be because not all of the PPYNP added per lipid to the JMs could be integrated into the micromotor structure. Thus, the PPYNP-to-lipid mass ratio of 1× was the upper limit for the preparation of JMs with (NIR) light stimulation. Based on these results, our studies were continued with the anisotropic and photothermal treatment (PTT)-responsive JPPs with the addition of ×1 PPYNP to prepare multiresponsive JMs in the following section. The photothermal conversion efficiency of the same PPYNP (×1) added to JPP3 and JPP4 was calculated and determined to be 64 ± 4.4% (see Figure 3). The reason for the low photothermal conversion efficiency of JPP3 and JPP4 with the same amount of PPYNP added was thought to be that part of the surface of the PPYNP covered with the polymer or lipid or was not fully incorporated into the Janus structure. To gain a deeper understanding, the microstructure of JPP3 and JPP4, which were prepared with PPYNP (×1), was examined using SEM, as shown in Figure 2. The SEM images were analyzed in detail, and the diameter and length distributions of JPP3 and JPP4 are shown in Figure S8. It was found that the structures did not have smooth surfaces and had projections on all surfaces.

### 3.4 Formation of MNP- and PPYNP-Loaded Multistimulus-Responsive JMs

Inspired by tadpoles, we combined the magnetic and NIR actuation properties of polymer-/lipid-based JMs to prepare a multistimuli-responsive functional micromotor system. We used fuel-free thermophoretic motion and a magnetic field to move microrobots and then investigated the influence of experimental parameters on the multistimulus-responsive behavior of micromotors. These multistimulus-responsive micromotor systems were prepared using MNP (×2) and PPYNP (×1) according to the lipid mass ratios. The diameter and length values of JPMP1, which was prepared using PVA, were approximately 6.00 ± 2.28 and 7.08 ± 2.53 μm (with an aspect ratio of ∼1.16 ± 0.12), respectively (Figure S14). In addition, the diameter of JPMP2, which was prepared using SDS as a surfactant with SDBS, was determined to be 4.99 ± 1.65 μm, and the length was measured to be 7.57 ± 3.62 μm (see Figure S14). Similarly, JPMP2 retained its asymmetric structure with an aspect ratio of nearly ∼1.41 ± 0.07.

**Figure 4:**
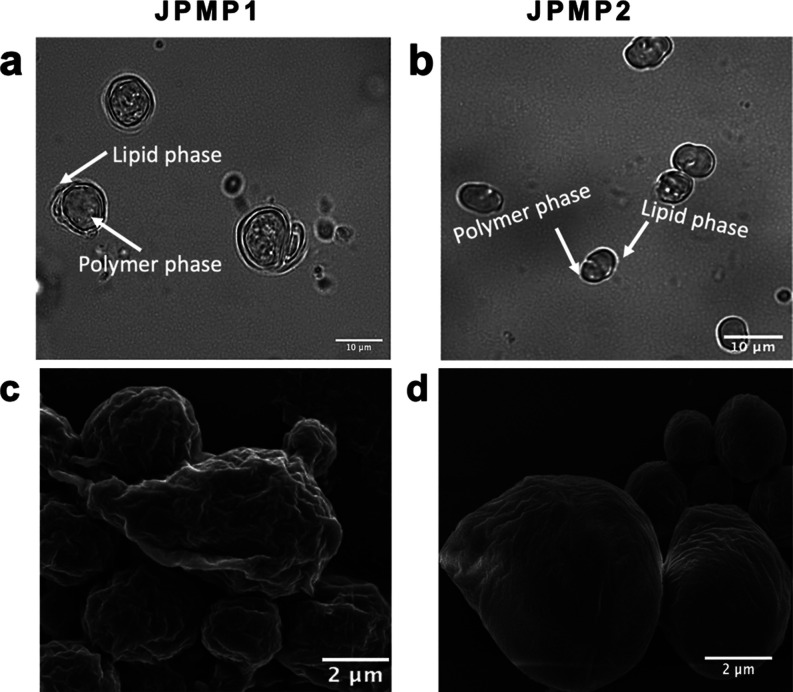
(a,b) Light and (c,d) SEM images of JPMP1 and JPMP2. JPMP1 with ×1 PPYNP and ×2 MNPs (external phase containing PVA/SDBS cosurfactants) JPMP2 with ×1 PPYNP and ×2 MNPs (external phase containing SDS/SDBS cosurfactants).

Figure 4 shows the light microscope and SEM images of the JMs. The addition of both MNPs and PPYNP into the Janus structure did not significantly affect the droplet and particle size of the micromotors; it is clearly observed that both particle formulations have an asymmetrical geometry like empty forms. It was determined by analyzing ∼30 particles using SEM images that ∼40% of JPMP1 and ∼60% of JPMP2 had an asymmetric geometry. The distributions of the diameter and length of JPMP1 and JPMP2 analyzed via SEM are shown in Figure S8. Besides that, we performed SEM–EDX analysis for detailed characterization of JMs and confirmed the presence of Fe in the particles (Figure S15).

We also performed FTIR analysis to verify the structures of the selected particles, as demonstrated in Figure S16. The results show that Precirol ATO 5, which is the lipid part of all types of particles, showed major peaks at 1721.18 cm^–1^ due to −C=O stretching and between 2914 and 2848 cm^–1^ due to −C–H stretching.^51^ It is important to note that PCL, which is the polymer part of each particle, also showed a carbonyl stretching peak at 1721.18 cm^–1^, causing the two bands to overlap. The peak at 1293.54 cm^–1^ is assigned to the backbone C–C and C–O stretching modes in the crystalline PCL. The peaks show asymmetric COC stretching vibrations (1238 cm^–1^), OC–O stretching vibrations (1218 cm^–1^), and symmetric COC stretching vibrations (1171 cm^–1^).^52^ The FTIR spectrum of PPY shows bands attributed to pyrrole ring stretching vibrations (1525.92 and 1446.37 cm^–1^), C–N stretching vibrations (1286.79 cm^–1^), aromatic ring bending vibrations (1150.35, 1026.92 cm^–1^), as well as C–C deformation (960.39 cm^–1^).^53^ Although the peaks originating from PPY in the 1460–900 cm^–1^ region cannot be clearly detected because PCL and Precirol ATO 5 have characteristic peaks in the structure, one of the significant peaks of PPY around 1540 cm^–1^, which is not present in the main JM structure and originates from the stretching vibration of the pyrrole ring, was observed in the FTIR spectrum of JPMP1 and JPMP2 particles. This confirms that PPYNPs are incorporated into the JM structure.

**Figure 5:**
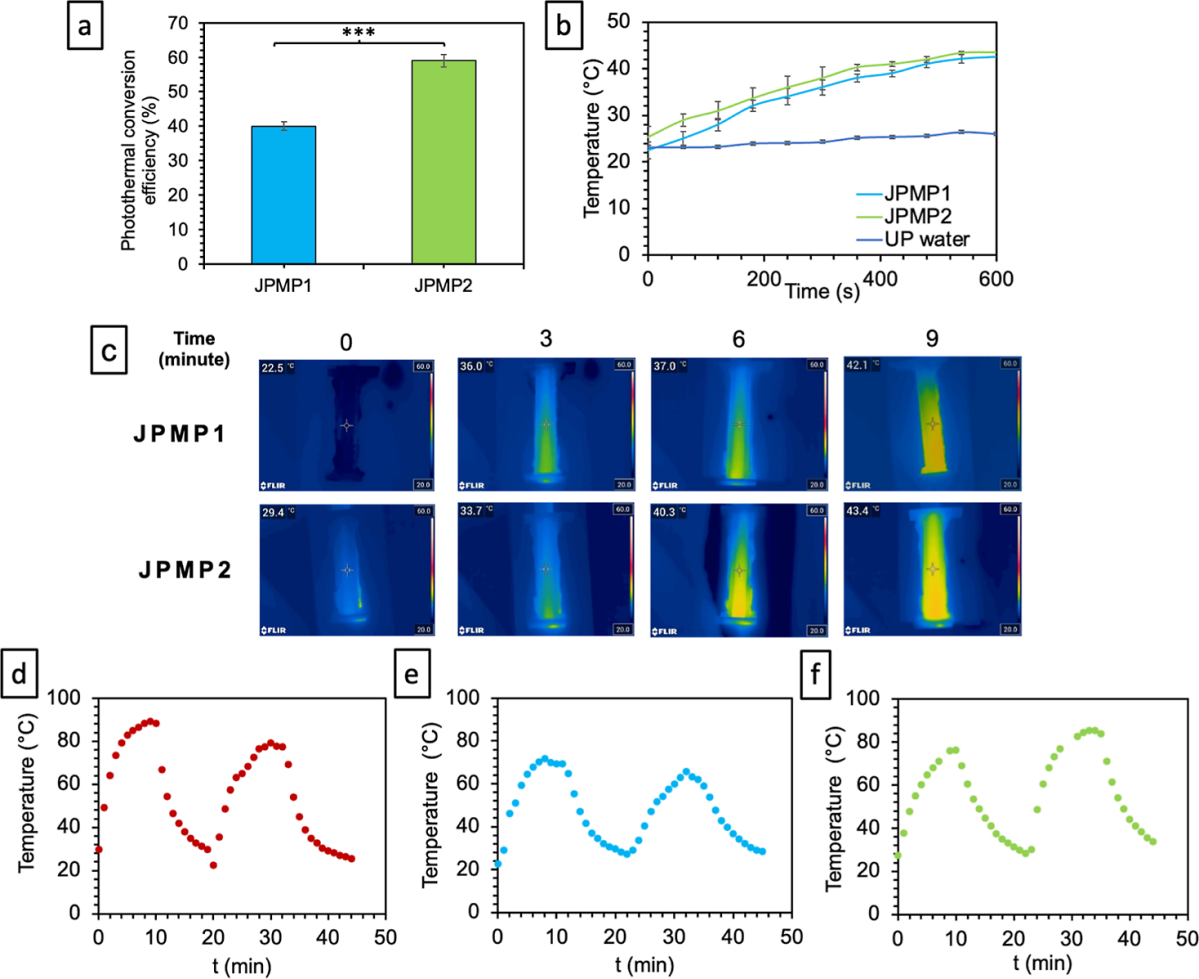
(a) Photothermal conversion efficiency (%). (b) Temperature change profiles in UP water. (c) Thermal camera images of the UP water channel containing JPMP1 and JPMP2 at 808 nm NIR irradiation and 2.5 W power, on–off cycles of (d) PPYNP, (e) JPMP1, and (f) JPMP2.

**Figure 6:**
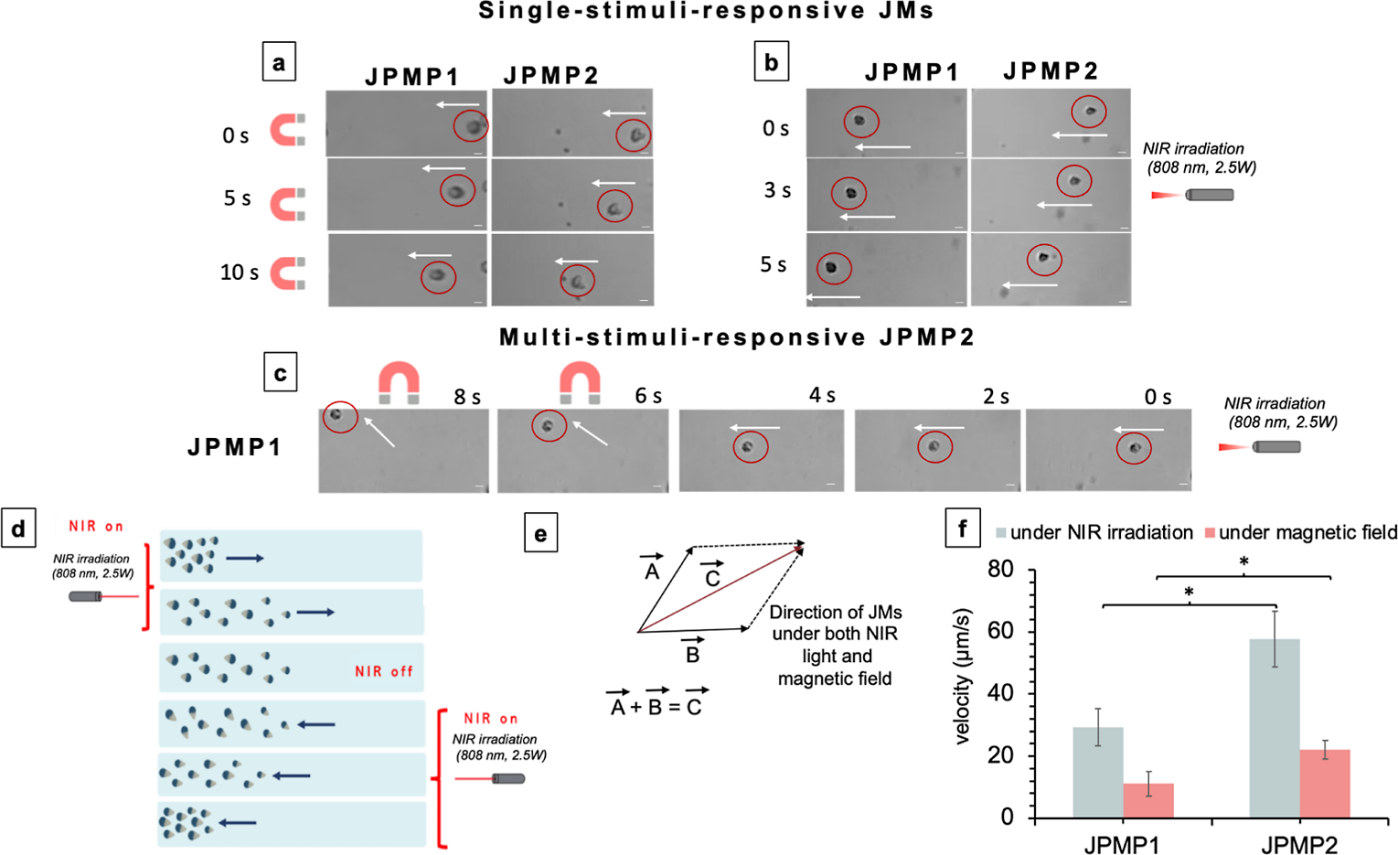
Movement of JPMP1 and JPMP2 under a (a) magnetic field (480 mT, see Movie S1 for JPMP1) and (b) 808 nm (2.5 W) NIR light irradiation. (c) Multistimuli-responsive abilities of JPMP2 under first 808 nm (2.5 W) NIR light irradiation and both NIR light irradiation and the magnetic field (480 mT). (d) Schematic illustration of on/off/on motion control of the polymer/lipid-based tadpole-like multistimuli JMs. (e) Schematic illustration of vectorial motion of JMs under NIR radiation and the magnetic field applied. (f) Velocity of JMs in UP water medium separately under NIR light irradiation and a magnetic field (480 mT). Scale bar = 10 μm. One-way ANOVA with Tukey’s post hoc test for multiple comparisons was used to assess data dispersion. Statistical significance was considered at *p < 0.05, **p < 0.01, and ***p < 0.001.

After verifying the structures, the photothermal performance of these multistimulus particles was evaluated first. The photothermal conversion efficiency values were calculated to be nearly 40 and 59% for JPMP1 and JPMP2, respectively, where JPMP2 gave the highest photothermal conversion efficiencies among the prepared JPs, as shown in Figure 5a. In addition, the temperature changes obtained from the dispersion of these JPs are presented in Figure 5b,c. Considering that the temperature change of UP water without JPs was nearly 3.2 °C (see Figure S13), the temperature of JPMP1 and JPMP2 increased from 22.1 to 42.1 °C and from 29.4 to 43.4 °C, respectively, within 10 min. Preclinical studies and clinical settings have observed that the tumor cells die at a high rate when exposed to temperatures above nearly 42 °C for 30 min^54^ A high temperature higher than 45 °C severely affects healthy cells, can cause inflammation, and can severely affect immune cells involved in antitumor immunity. The fact that the temperature did not rise to this level clearly showed that the prepared JPMP1 and JPMP2 could be easily used as PTT-responsive micromotors. To evaluate the photothermal stability of PPYNP (×1), JPMP1, and JPMP2, which were essential assessment parameters in PTT, the particles were irradiated under 808 nm laser for 10 min; then the system was allowed to cool down to the initial temperature, and the on–off cycles were repeated two times (Figure 5d–f for PPYNP, JPMP1, and JPMP2, respectively). After two cycles of the on–off process, an efficient photothermal response of PPYNP, JPMP1, and JPMP2 displayed a similar performance in this cyclic examination. Besides that, to prove both the single and multiresponsive properties of JPMP1 and JPMP2, the trajectories and velocities of the magnetic and NIR-driven micromotors were analyzed in UP water. First, the JMs, injected into the UP water medium, were actuated only under a 480 mT magnetic field. Figure 6a illustrates the trajectories of the magnetically actuated JMs in water. When the speeds of JPMP1 and JPMP2 were analyzed, their motion speeds were calculated to be 11 ± 4 and 22 ± 6 μm/s (Figure S17A) (Movies S1 and S2, respectively). The JMs were motionless without a magnetic field and an NIR laser.

Subsequently, after injection of the JMs into the UP water medium, only NIR light (808 nm, 2.5 W) was applied to the micromotors to investigate the NIR stimulus response of the JMs. When the NIR light (808 nm, 2.5 W) was irradiated, the JMs started to move due to the photothermal effect of the PPYNP.^37^ Here, a temperature gradient was formed between the nonirradiated and irradiated surfaces of the JMs, and the temperature gradient caused the microrobot to move. The movement was in the opposite direction of the light, as pointed out in the literature.^37^ When NIR light was applied from the left side of the channel, the micromotors were moved to the right of the channel. The trajectories of the NIR-driven JMs in the water are shown in Figure 6b. The velocities of JPMP1 and JPMP2 were measured as 29 ± 6 and 57 ± 9 μm/s (Figure S17B), as demonstrated in Movies S3 and S4, respectively. As can be clearly seen from the results, the prepared microrobot systems can be stimulated separately under both a magnetic field and NIR light.

To demonstrate the multiresponsive capabilities of JMs, JPMP1 was selected as an example, and the movement trajectory of both the magnetic and NIR-driven JPMP1 was also investigated in UP water (Figure 6c). First, JPMP1 was injected into the channel containing UP water and irradiated with a NIR light (808 nm, 2.5 W). The micromotors started to move in the opposite direction of the NIR light as the temperature increased with the NIR light irradiation (see Figure 6c). After 4 s, the NIR irradiation was combined with a magnetic field from the side (top) of the channel, and the behavior of the microrobots was investigated, as shown in Figure 6c (Movies S5 and S6 show the multistimulus behaviors of JPMP1 and JPMP2). As can be clearly seen from the figure, the micromotors showed vectorial motion under the NIR radiation and applied a magnetic field from the side of the channel instead of following a linear directional motion (Figure 6e). In conclusion, the obtained data again emphasized that functional JMs were clearly responsive to both NIR light and the magnetic field.

The “on/off” movement of these polymer/lipid-based tadpole-like JMs could be controlled by the “open/close” mode of the NIR light source (as shown in Figure 6d). JPMP2 was selected as an example to investigate this phenomenon. JPMP2 demonstrated the reversible starting and stopping of the prepared JMs when the NIR laser was switched on/off (Figure 6d). Then, the micromotor returned to the original position by applying a NIR light (808 nm, 2.5 W) in the opposite direction. An illustration of the “on/off” motion control of JPMP1 and JPMP2 by NIR laser irradiation in the water medium is given in Movies S7 and S8, respectively. The application and discontinuation of the NIR laser enabled on–off motion control for both JPMP1 and JPMP2.

Finally, to compare the propulsion performance of the two JMs systems, we calculated the velocity of the microrobots, as shown in Figure 6f. Although JPMP1 and JPMP2 exhibited multistimulus-responsive properties and could move successfully in water, the fastest of the JMs was JPMP2, with an aspect ratio of ∼1.41, compared to JPMP1, with an aspect ratio of ∼1.16. All of these indicate that the Janus-structured organic micromotor particles have excellent responsiveness to multiple stimuli and desired controllability.

### 3.5 Catalytic Activity of Multistimuli-Responsive JMs for the Degradation of Methylene Blue

The choice of MNPs was to give the JMs not only the magnetic manipulation ability but also the catalytic activity properties of the JMs, which comes from the Fenton reaction-forming capability of Fe-containing MNPs. MNPs stabilized by oleic acid and hydrophobic in nature can be used in the oxidative degradation of pollutants via the heterogeneous Fenton process. The Fenton reaction can be used to decompose various chemicals such as MB, Red 120, Red MX-5B, Reactive Yellow 84, etc.^55,56^ Since the hydroxyl radical (^•^OH) is directly responsible for the oxidative degradation in Fenton reactions, the degradation rate of dyes is determined by the generation rate of the ^•^OH and the decomposition rate of H_2_O_2_.^57^^•^OH can also be used in the chemodynamic therapy of the various types of cancer by themselves or in synergistic therapy studies, a highly studied topic recently.^58^

Many studies underlined that the Fe_3_O_4_ NPs show high stability and catalytic activity under different environmental conditions, and the optimal pH for Fe_3_O_4_ NPs catalysis was around 3.^59,60^ Therefore, at these pH values, the MB degradation efficiency increases due to the formation of water and oxygen by the decomposition of hydrogen peroxide and the formation of ^•^OH by the Fe^2+^ ion.^60^ However, as the reaction proceeds, the Fe_3_O_4_ NPs have a tendency to clump together, forming larger aggregates. Moreover, the Fe^2+^ leaching into the solution limits their ability to disperse effectively, reducing their primary larger specific surface area and their stability for subsequent reuse. So, it is important to find a suitable carrier capable of promoting the effective dispersion of Fe_3_O_4_ NPs and guaranteeing the preservation of the catalyst.^56^

**Figure 7:**
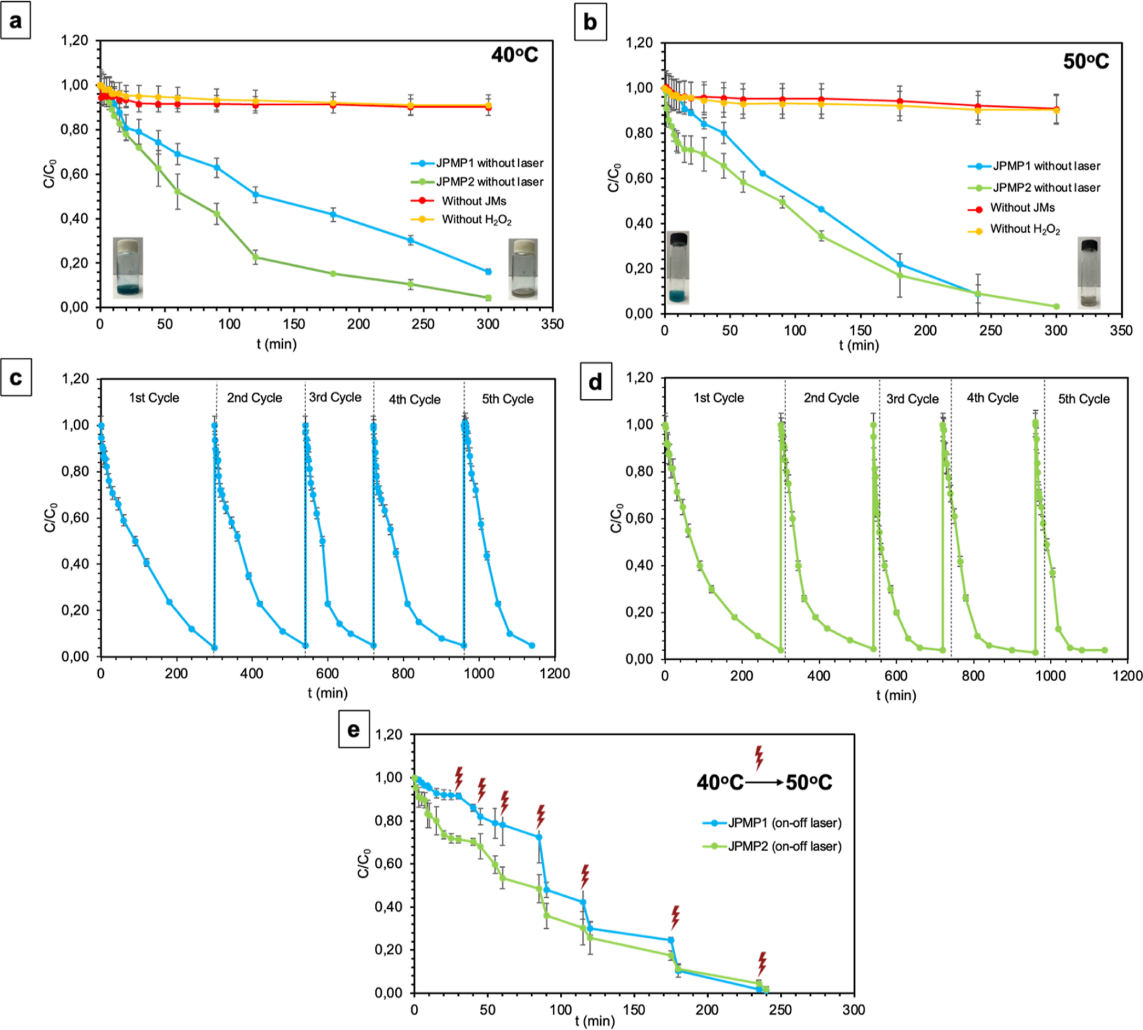
Degradation of MB over laser time at pH 2.5 under different temperature conditions: (a) 40 and (b) 50 °C. The recyclability of (c) JPMP1 and (d) JPMP2 at 40 °C. (e) 40–50 °C heating upon NIR laser irradiation at on–off mode.

Considering all these, the purpose of exploring the properties of new JMs, which were heterogeneous Fenton catalysts, was not only to determine their catalytic performance by protecting Fe_3_O_4_ NP stability but also to throw light on the various application areas of multifunctional JMs. For this purpose, the decomposition rate of H_2_O_2_ was analyzed at two different temperature values, 40 and 50 °C at pH 2.5, considering the importance of the effect of temperature on heterogeneous Fenton reactions and the fact that the catalytic efficiency drops drastically when the pH is higher than 3.^60^ Since selected Precirol ATO 5, which has a melting point of 54–59 °C, undergoes a solid–liquid phase transition above 54 °C, the Fenton reaction was studied below this value. The catalytic degradation of MB by JPMP1 and JPMP2 is demonstrated in Figure 7a,b. The degradation of MB as a function of reaction time at pH 2.5 in the absence of H_2_O_2_ or in the absence of JMs was also investigated. It was observed that MB degradation did not occur because Fe^2+^ could not catalyze the reaction for the formation of ^•^OH radicals in the absence of H_2_O_2._ In addition, MB removal did not occur when no micromotors were added, which means the absence of Fe^2+^.^61^ These findings suggest that the discoloration of MB was primarily due to a heterogeneous Fenton-like reaction catalyzed in the presence of JPMP1 and JPMP2, which contain Fe^2+^ in their structure (see Figure 7a,b).

First, at 40 °C, the complete significant reduction at MB concentration was obtained within 300 min using JPMP1 and JPMP2, as shown in Figure 7a. However, after the initial rate period of the reaction (nearly 30 min), the degradation rate for JPMP1 was slower than that of JPMP2. The MB removal efficiency of JPMP1 was ∼31% within 1 h, whereas JPMP2 showed an enhanced removal ratio of MB of ∼28% within only 30 min. The MB removal efficiency of the JPMP1 reached 85% at the end of the third hour, while JPMP2 greatly enhanced the removal ratio to 96%. The catalytic performance of JPMP1 and JPMP2 was analyzed for the decolorization of MB at slightly higher pH values, such as pH 2.5 (Figure S18). When JPMP1 and JPMP2 treated the MB solutions, as the reaction progressed, the color of the MB solution gradually faded, indicating a significant reduction in MB concentration. This occurrence can be attributed to the destruction of the entire molecule.^62^

Although JPMP1 and JPMP2 showed similar profiles in the Fenton reaction and degraded MB at almost similar times, the ability of JPMP2 to degrade MB was slightly more extraordinary for MB rather than JPMP1 at 40 °C. The reason for this is related to the positioning of the MNP included in the particle structure within the lipid and polymer compartments of the Janus architecture, guided by their physicochemical properties. In the literature, it is emphasized that a molecule with a hydrophilic nature will be located within the more hydrophilic lipid compartment (Precirol ATO 5), while a hydrophobic molecule will be located within the more hydrophobic polymer compartment (PCL). In light of this information, due to the hydrophobic characteristics provided by the lipid coating (oleic acid) as well as the physicochemical properties of MNPs, it is evaluated that MNPs are located in both the lipid and polymer compartments. Given this insight, the position of the compartments in the Janus structure and its contribution to the asymmetry are crucial. The lipid compartment of JPMP1, compared to JPMP2, may serve as a buffer layer to achieve the maximal Fe^2+^ concentration (see Figure 4). Considering the thick lipid layer of JPMP1, the amount of Fe leached may be quite small. This actually makes it clear that the loading capacity on JMs can also be adjusted by changing the proportions of the compartments and other processing conditions. A similar situation is observed in studies of hydrophobic and hydrophilic drug release from JPs. Previous studies in our group showed that the release profiles of hydrophilic and hydrophobic drugs were affected by the positions of the lipid compartment and the asymmetry. Hydrophilic drugs are released more rapidly from structures with a larger aspect ratio due to the elongation of the lipid compartment, while drug release occurs more slowly from structures with a smaller aspect ratio due to reduced surface area.^27^ On the other hand, while the polymer compartment offers a high hydrophobic drug loading capacity, the lipid compartment wraps around the polymer in a thin layer to ensure maximum drug molecule retention inside the polymer compartment.^27,63^

In light of this information, JPMP2, which has a higher aspect ratio than JPMP1, is expected to exhibit a higher interaction rate with MB, as the surface area of the Janus architecture will increase as the length of the lipid compartment increases. The literature emphasizes that the adsorption ability of materials depends on their physical and chemical properties as well as the surface area of the adsorbent, which is responsible for the mass transfer rate.^64^ Thus, the high surface area of JPMP2 can be associated with the high degradation efficiency of MB compared to JPMP1. After that, the degradation efficiency of the Fenton catalysts (JPMP1 and JPMP2) as a function of time at 50 °C was examined, as given in Figure 7b. Significant decolorization of the MB dye with both particles was achieved in 300 min at 50 °C, but their performance was closer compared to 40 °C (see Figure S19). Both particles exhibited an ∼91% MB removal rate at the end of the fourth hour, even though the removal of MB by JPMP2 appeared to be faster compared to JPMP1 during the first 4 h period. As mentioned, the melting temperature of the Precirol ATO 5 is close to 50 °C, so at this temperature, the soft nature of the lipid part erased the effect of the geometry (aspect ratio) between particles.

The reusability of JPMP1 and JPMP2 catalytic micromotors was investigated by recycling the particles to evaluate their catalytic performance. It is clearly seen in Figure 7c,d. The catalytic activity of JPMP1 and JPMP2 was maintained even after five reaction cycles, pointing to the long-term stability and reusability of the micromotors. It is confirmed that the MB degradation efficiency of both particles remained stable during the consecutive 5 cycles. The decolorization of the solution with time is presented in Figure S18, from which it can be clearly seen that the color of the MB changed from blue to transparent at the end of the reaction. In addition, upon completion of the reusability experiments, at the end of 5 cycles, JPMP1 and JPMP2 were imaged using a light microscope, and their response to the magnetic field was also investigated as a control experiment. As can be seen from Figure S21, it was found that the particles maintained their structural integrity; however, their velocity showed a slight decrease, which is less than 40%.

In addition, NIR irradiation was applied to JPMP1 and JPMP2 in each measurement during the Fenton reaction at 40 °C, and the temperatures of the samples were increased to 50 °C by irradiation of the NIR laser, as shown in Figure 7e. The temperature elevation of both JMs is achieved owing to the high NIR light-absorbing property of PPYNP, which has a high photothermal conversion efficiency under NIR radiation (808 nm). The temperature change can affect how JMs interact with their environment and respond to external stimuli due to their special structures as well as affect the reaction rate in reaction types that require special environmental conditions, such as the Fenton reaction. In light of this information, it was aimed to analyze how the performance of JMs was affected during the Fenton reaction by increasing the temperature using an external effect. As shown in Figure 7e, NIR applied within the first 1 h did not cause any significant change when compared to MB removal profiles without NIR. After 1 h of the reaction, NIR radiation caused an increase in temperature and triggered a reaction, and an increase in MB degradation was seen for both JPMP1 and JPMP2. Even if the removal of MB by JPMP2 appeared to be faster than that by JPMP1 during the experiment, the degradation ratio of MB was determined as ∼98% for both JMs at the end of the experiment. As previously mentioned, the asymmetry in the structure of JPMP2, as opposed to that of JPMP1, impacts the removal of MB. This is because the lipid compartments alter the surface area due to their variations in positioning and elongation between the structures. This change will affect the rate at which JMs interact with MB; it consequently leads to changes in their removal efficiencies. Additionally, considering the responses of JPMP1 and JPMP2 to the magnetic field and NIR radiation (see Figure 6f), despite their exhibiting similar profiles in the Fenton reaction and nearly simultaneous completion of the reaction, JPMP2’s higher capacity for removing MB compared to JPMP1 can also be explained. To conclude, JMs can be used as a heterogeneous Fenton catalyst for the degradation of various organic pollutants and decolorization without external energy.

**Figure 8:**
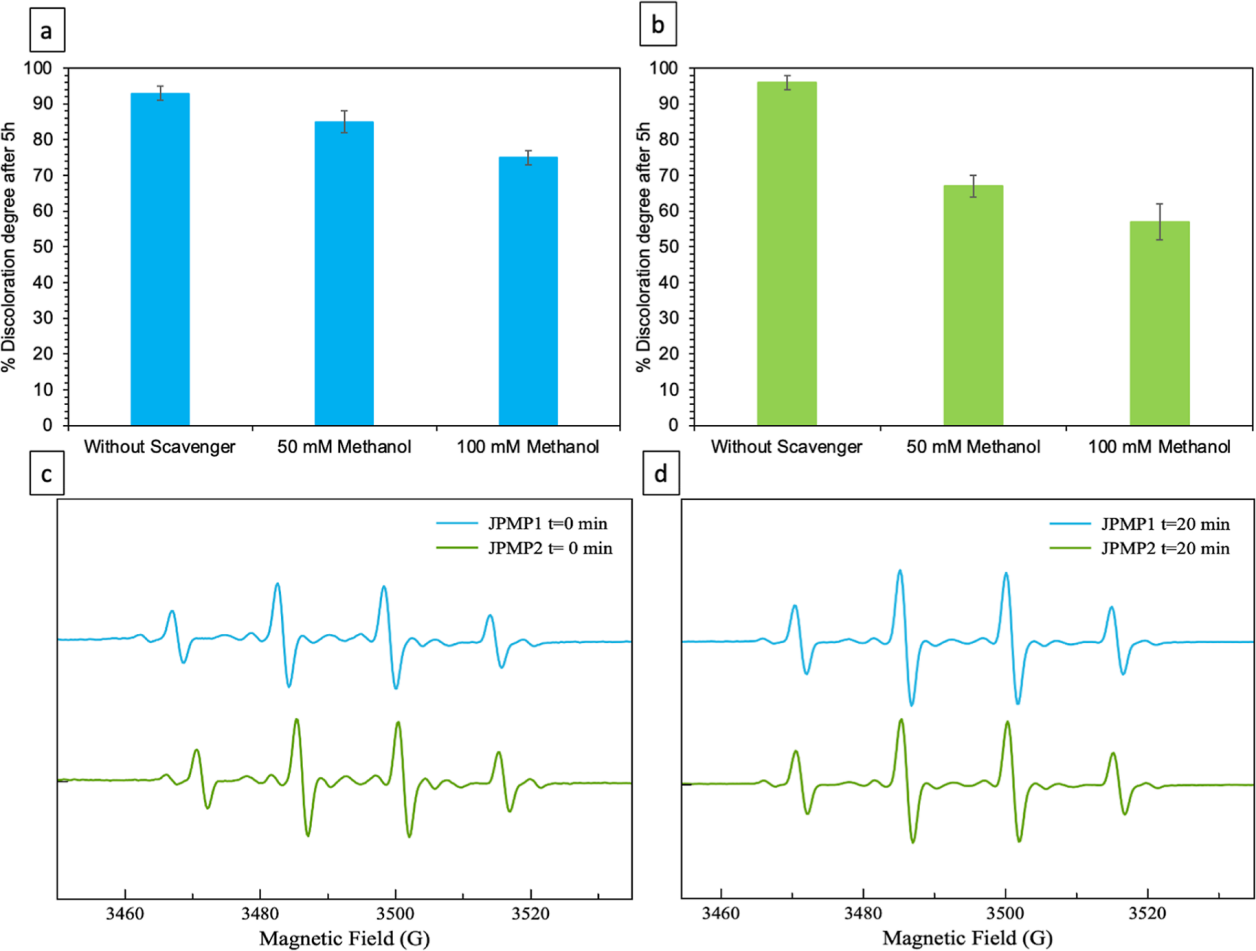
Effect of radical scavengers on the removal of MB in the presence of (a) JPMP1 and (b) JPMP2. Typical EPR spectra with the presence of DMPO-^•^OH adducts for JPMP1 and JPMP2 systems at (c) 0 and (d) 20 min.

To determine the mechanism behind the MB removal and to confirm the presence of radicals, the effect of the free radical scavenger, methanol, on the MB removal was investigated (see Figure 8a,b for JPMP1 and JPMP2). It can be seen from Figure 8a that the percentage of discoloration in the presence of JPMP1 decreased by 9% (down to 85%) when 50 mM methanol was used and decreased by 19% (down to 75%) when the concentration of methanol was increased to 100 mM. It can be seen from Figure 8b that the percentage of discoloration in the presence of JPMP2 decreased by 30% (down to 67%) with 50 mM methanol and decreased by 41% (down to 57%) when 100 mM methanol was added. The methanol was used as a scavenger for ^•^OH radicals, and significant inhibition of the MB degradation reaction was observed. Furthermore, EPR analysis was employed to deeply explore the contribution of ROS generated by JMs in MB degradation. DMPO was used as a trapping agent, and EPR analysis was employed to form spin adducts DMPO-^•^OH. EPR spectra of the JPMP1/JPMP2–H_2_O_2_–DMPO system recorded at different times, with the same conditions, are given in Figure 8c,d. As shown in these figures, the EPR spectra were composed of quartet signals separated by ∼15 G with relative intensities of 1:2:2:1, which is the characteristic signal of DMPO-^•^OH spin adduct, proving the generation of ^•^OH.^20^ Therefore, the radicals reported by the scavenging experiments are consistent with the types of ROS detected by the EPR study performed for the JPMP1 and JPMP2 systems (Figure 8).

To understand in depth whether the slight differences between JPMP1 and JPMP2 are due to the positioning of the compartments in the structure of the JMs, hydrophilic Rhodamine B, which is a fluorescent hydrophilic dye, was used to study the localization within JPMP1 and JPMP2. It is given in the literature that polymer/lipid Janus structures, as studied here, have the ability to encapsulate molecules with higher hydrophobicity to the polymer part and less hydrophobic/and more hydrophilic molecules to the lipid part.^63^ The microscopic images of JPMP1 and JPMP2 containing Rhodamine B are given in Figure S22. As can be clearly seen from these images, the hydrophilic dye was selectively encapsulated by some portion of the JMs, which was proposed to be the lipid part.

### 3.6 In Vitro Cytotoxicity Assay of JPMPs

**Figure 9:**
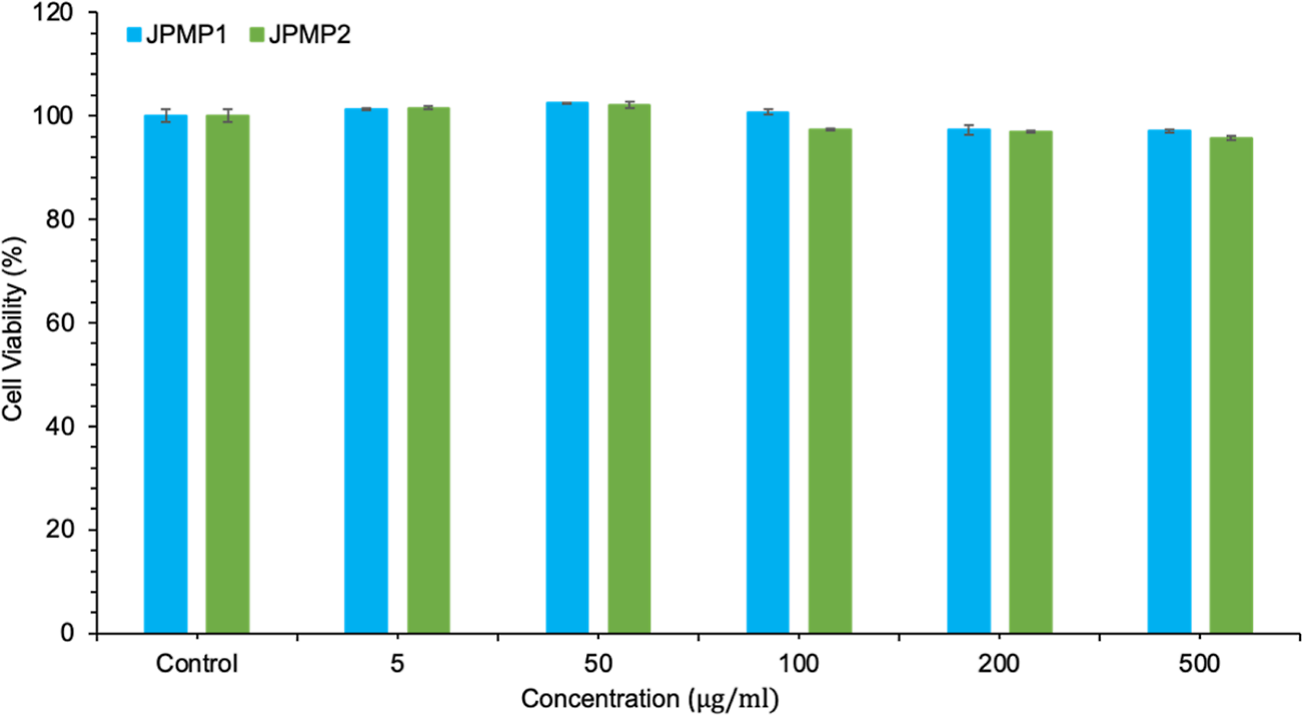
In vitro cytotoxicity study of L-929 (mouse fibroblast cell line) after incubation with JPMP1 and JPMP2 for 24 h was determined by MTT assays (mean ± SD, *n* = 3 independent formulations).

The L-929 assay was used to evaluate the cytotoxicity of JPMPs. To evaluate the effect of JPMPs on cell viability, various concentrations of JPMPs were added to the cell cultures. The MTT assay was used for the evaluation of the cytotoxicity after 24 h of incubation. The results in Figure 9 demonstrate the cell viability associated with increasing concentrations of JPMPs. The data showed that the L-929 cell line exhibited high survival rates after exposure to PCL/Precirol ATO 5-based JPMPs.^65^ These observations confirmed that the developed JPMP formulations can be safely used for drug delivery applications at concentrations up to 500 μg/mL and 24 h exposure.

## 4 Conclusions

In our study, we present multistimulus-responsive polymer and lipid-based JMs, which were prepared using PPYNP and magnetic NPs. Based on the Marangoni effect, the Janus microrobots can move in liquid environments and move up, down, left, and right induced by the temperature difference. With the ability to respond to multiple stimuli, Janus microrobots can achieve synergistic motion via magnetic or NIR light stimulation. As a new alternative for developing multienergy field-driven microrobots, this multifunctional motion feature can improve the working ability and efficiency of the microrobot. The JMs with asymmetric geometry will improve future research priorities and applications in intelligent on–off control systems with biodegradability of their manufacturing materials, large-scale producibility, and the ability to overcome various biological barriers via tunable functions. Furthermore, the multicompartment JPs can also transport therapeutic agents as delivery platforms. As a result, they can be utilized as versatile materials for actively controlled motion, drug delivery, biosensing, and imaging, with significant potential in the field of precision medicine. Importantly, the degradation mechanism of MB by JMs with the Fenton reaction was proposed for the first time in our study. JMs are also potent Fenton catalysts for the degradation of various organic pollutants and decolorization without external energy.

## Supporting Information Available

Fabrication procedure of JMs; physicochemical properties and composition of JMs; characterization of oleic acid-coated magnetic NPs; calibration curves of MB; surface tension values of the external phases; size distribution of JP1, JP2, JPM1, JPM2, JPM3, JPM4, JPP1, JPP 2, JPP3, JPP4, JPP5 and JPP 6, JPMP1, and JPMP2; light microscopy images of JPPs prepared by using different ratios of PPYNP; thermal camera images to observe temperature change in UP water at 808 nm NIR irradiation and 2.5 W power; EDX Analysis of JPMP1 and JPMP2; FTIR spectra of JP1, JP2, JPM3, JPM4, JPMP1, JPMP2, and PPYNP; photothermal conversion efficiency calculation; velocity distribution of magnetic and NIR-driven JPMP1 and JPMP2; visual inspection of the color change of the MB solution before and after a Fenton reaction; characterization of JPMP1 and JPMP2 micromotors at the end of 5 cycles of Fenton reaction; and optical and fluorescence microscopy images of JPMP1 and JPMP2 (PDF)
Motion of JPMP1 under a magnetic field in a water medium (MP4)
Motion of JPMP2 under a magnetic field in a water medium (MP4)
Motion of JPMP1 under a NIR irradiation in a water medium (MP4)
Motion of JPMP2 under a NIR irradiation in a water medium (MP4)
Motion of JPMP1 under both magnetic field and NIR irradiation in a water medium (MP4)
Motion of JPMP2 under both magnetic field and NIR irradiation in a water medium (MP4)
On/off motion of JPMP1 controlled by NIR irradiation in a water medium (MP4)
On/off motion of JPMP2 controlled by NIR irradiation in a water medium (MP4)
