# Supplementary material for: Multi-Stimuli-Responsive Tadpole-like Polymer/Lipid Janus Microrobots for Advanced Smart Material Applications
Source: ACS Appl Mater Interfaces. 2024 Feb 15;16(12):15533–47. doi: 10.1021/acsami.3c18826 (PMC10983008; doi:10.1021/acsami.3c18826)
Supplement: Supplementary file 1 — am3c18826_si_001.pdf [file am3c18826_si_001.pdf]

Supporting Information

A Multi Stimuli-Responsive Tadpole Like Polymer/Lipid Janus Microrobots for Advanced Smart Material Applications

Burcu Okmen Altas[1], Cansu Goktas [1,⊥], Guliz Topcu[1,⊥], and Nihal Aydogan[1,*]

[1]Department of Chemical Engineering, Hacettepe University, Beytepe 06800, Ankara, Turkey

[*]Corresponding Author: Nihal Aydogan

Fax: +90 312 2992124,

Phone: +90 312 2976781

E-mail:anihal@hacettepe.edu.tr

[⊥]Equal contribution at investigation

Video S1. The motion of the JPMP1 under a magnetic field in water medium

Video S2. The motion of the JPMP2 under a magnetic field in water medium

Video S3. The motion of the JPMP1 under a NIR irradiation in water medium

Video S4. The motion of the JPMP2 under a NIR irradiation in water medium

Video S5. The motion of the JPMP1 under both magnetic field and NIR irradiation in water medium

Video S6. The motion of the JPMP2 under both magnetic field and NIR irradiation in water medium

Video S7. The on/off motion of the JPMP1 controlled by the NIR irradiation in water medium

Video S8. The on/off motion of the JPMP2 controlled by the NIR irradiation in water medium

Spreading Coefficient Theory

The Janus microparticle structure prediction was calculated via 'Spreading Coefficient Theory' according to the following equation [1, 2];

$$S_i = \gamma_{jk} - (\gamma_{ij} + \gamma_{ik})$$ ……………………………..Eq. S1

Where i, j, and k represent the oil, water, and polymer phases, respectively.

S1 < 0; S2 < 0; S3 > 0 → core-shell morphology

S1 < 0; S2 < 0; S3 < 0 → acorn-shaped morphology

S1 < 0; S2 > 0; S3 < 0 → droplet separation

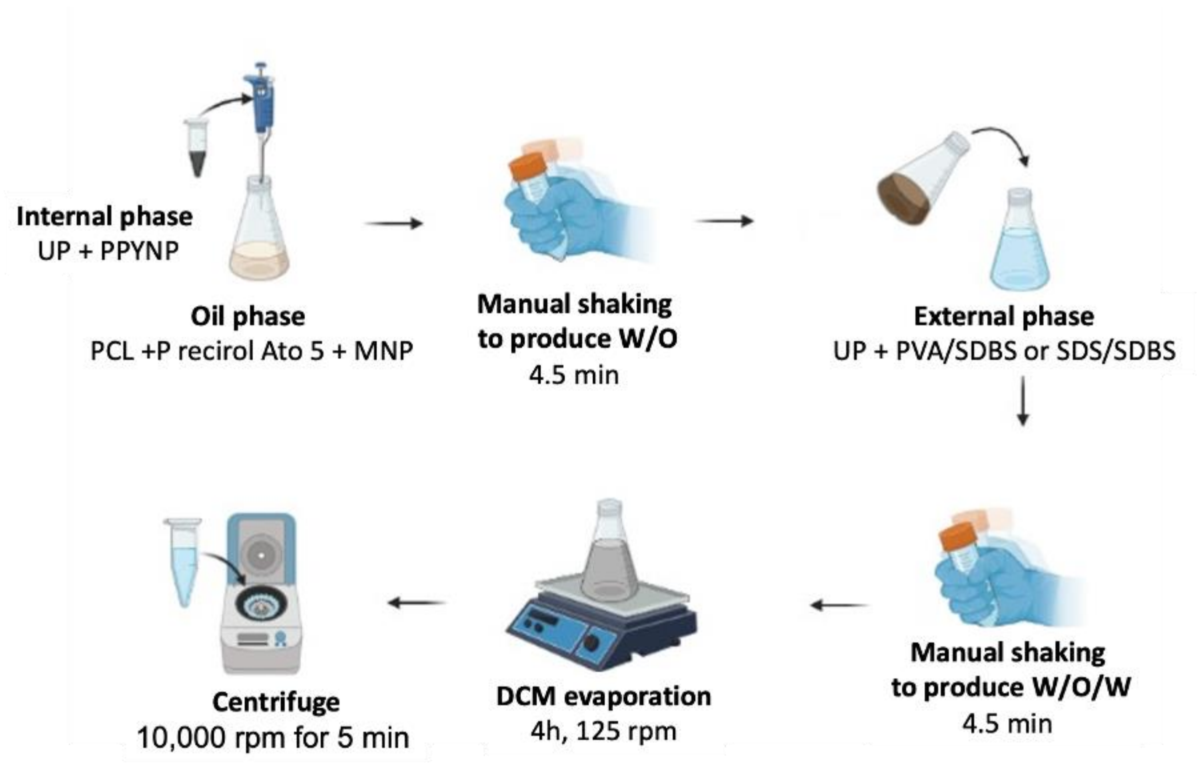

Figure S1. The fabrication procedure of Janus micromotor particles (JPMPs) (Created with BioRender.com)

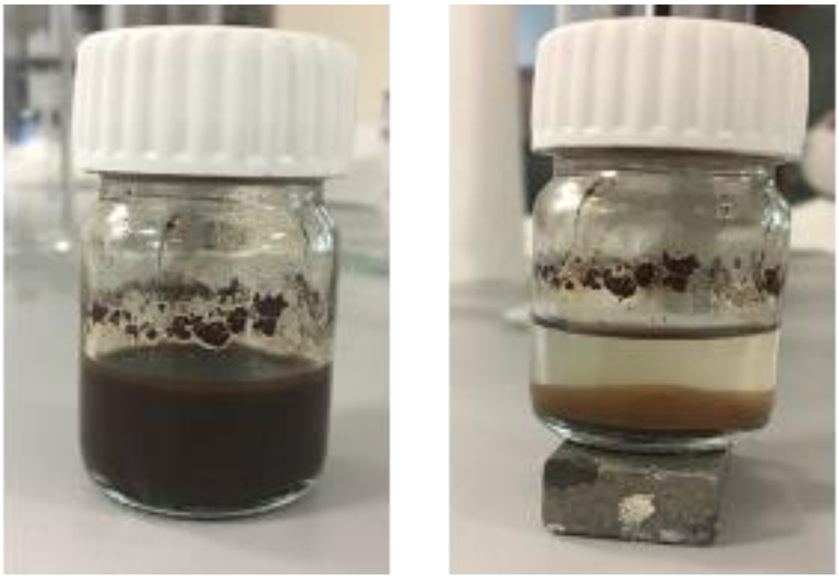

Figure S2. Oleic Acid coated magnetic nanoparticles

Table S1. Physicochemical properties and composition of the designed JMs (average±SD).

Code	*Surfactant*	*MNP*	*PPYNP*	*Droplet (μm)*	*Diameter (μm)*	*Length (μm)*
JP1	PVA/SDBS	-	-	14.68±2.04	6.59±1.80	7.89± 2.53
JP2	SDS/SDBS	-	-	12.60±1.96	8.49±1.96	14.19±4.41
JPM1	PVA/SDBS	×1	-	13.33±2.63	5.73±1.16	6.60± 1.67
JPM2	SDS/SDBS	×1	-	15.07±2.05	6.82±2.15	10.13±3.05
JPM3	PVA/SDBS	×2	-	13.76±2.38	7.18±1.81	8.75±2.06
JPM4	SDS/SDBS	×2	-	16.78±1.84	5.76±1.15	7.71± 2.21
JPM5	PVA/SDBS	×3	-	15.70±1.80		
JPM6	SDS/SDBS	×3	-	16.45±2.08	-	-
JPP1	PVA/SDBS	-	×0.5	13.76±3.35	7.20±1.69	9.43± 2.49
JPP2	SDS/SDBS	-	×0.5	12.93±1.95	5.90±1.33	9.57± 2.07
JPP3	PVA/SDBS	-	×1	13.85±1.80	8.17±3.19	9.50± 2.24
JPP4	SDS/SDBS	-	×1	14.72±2.29	5.93±0.87	9.08±1.02
JPP5	PVA/SDBS	-	×1.5	11.53±2.07	7.16±1.94	9.12± 2.14
JPP6	SDS/SDBS	-	×1.5	12.69±2.58	5.67±0.90	8.80± 2.07
JPMP1	PVA/SDBS	×2	×1	12.07±2.12	6.00±2.28	8.08±2.53
JPMP2	SDS/SDBS	×2	×1	13.18±0.99	4.99±1.65	7.57±3.62

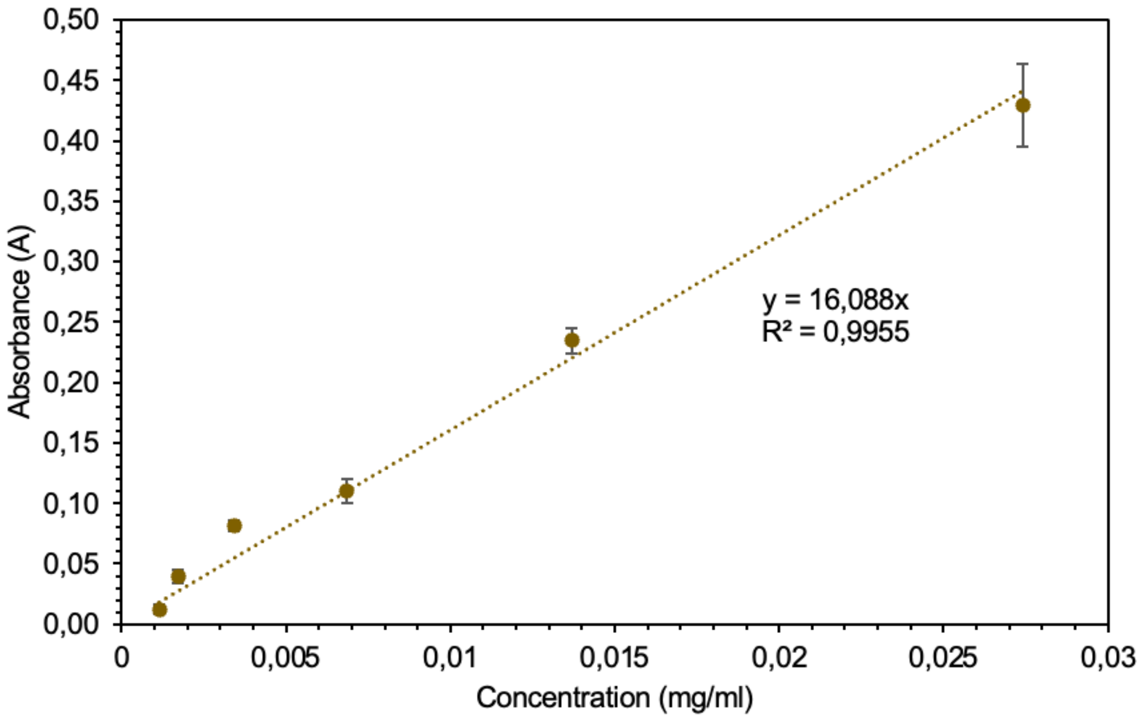

Figure S3. Calibration curves of methylene blue, y=16,088x

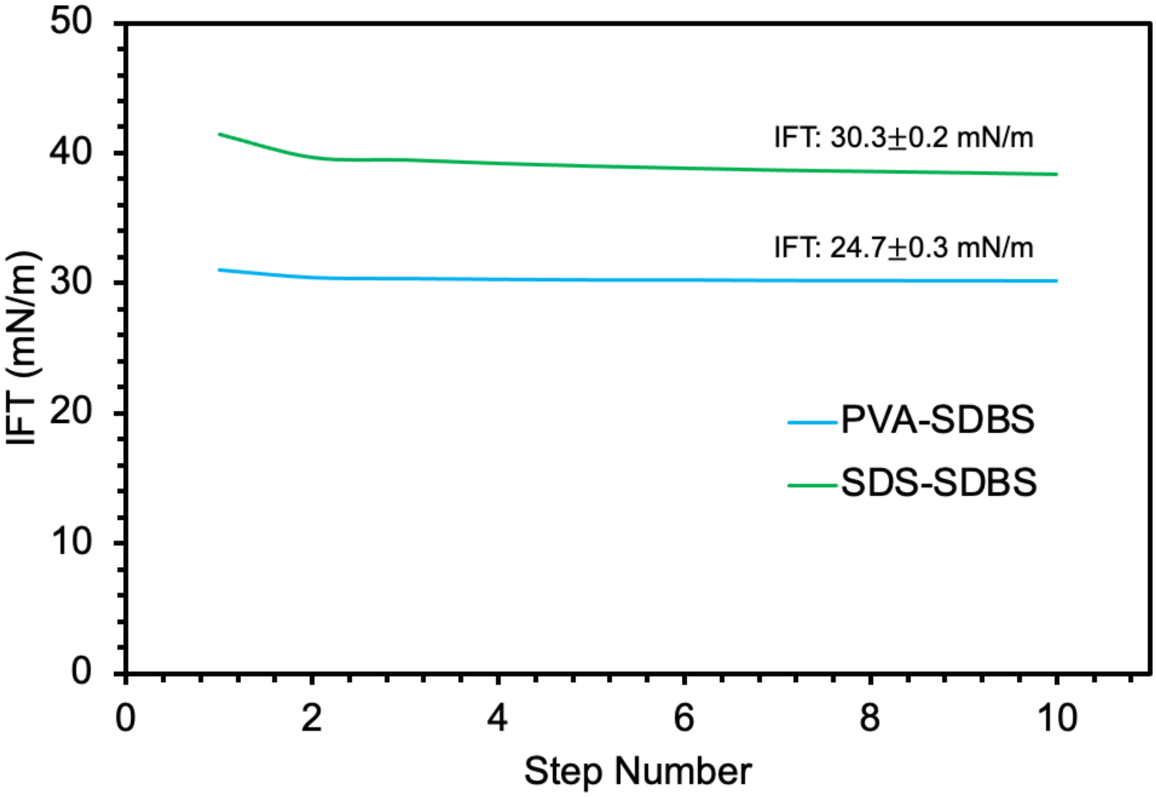

Figure S4. The surface tension values of the external phases

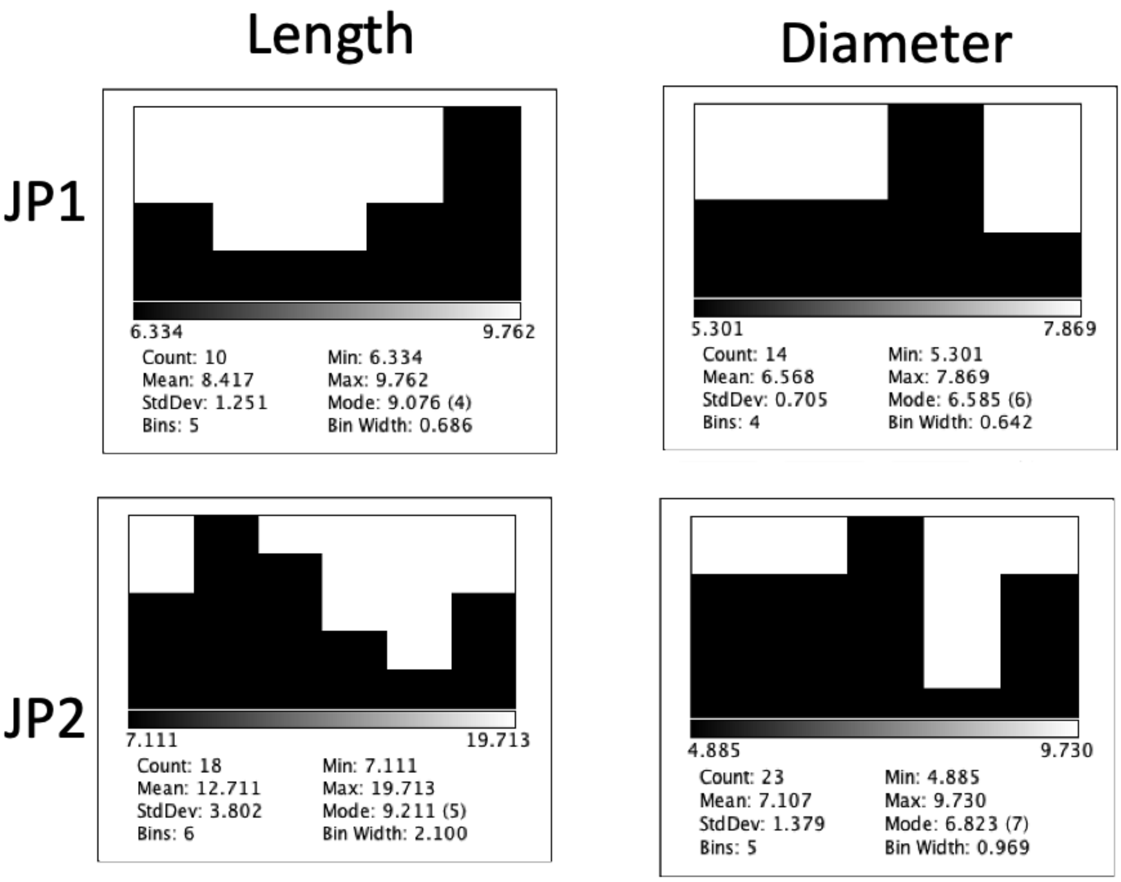

Figure S5. Distribution of diameter and length of JP1 and JP2.

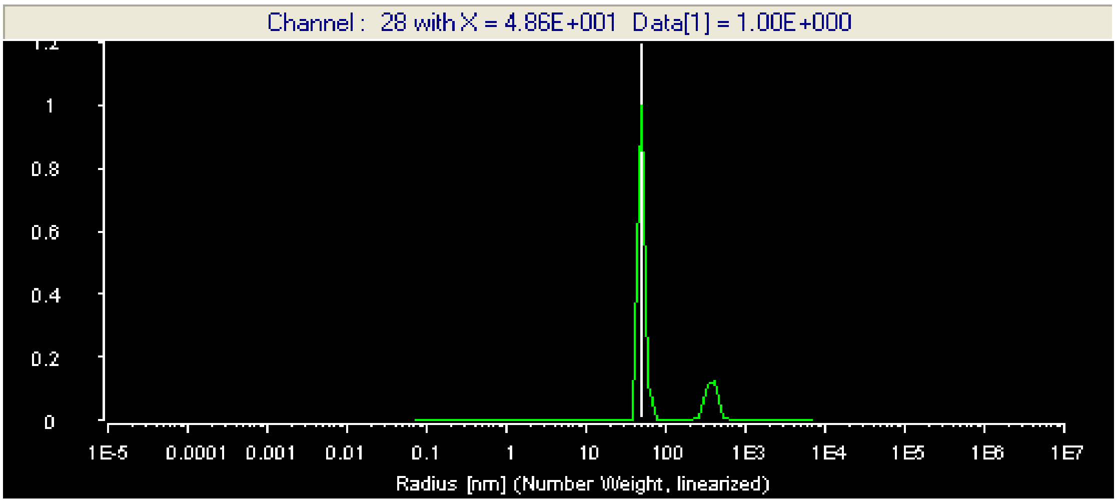

Figure S6. The hydrodynamic radius of oleic acid-coated MNP

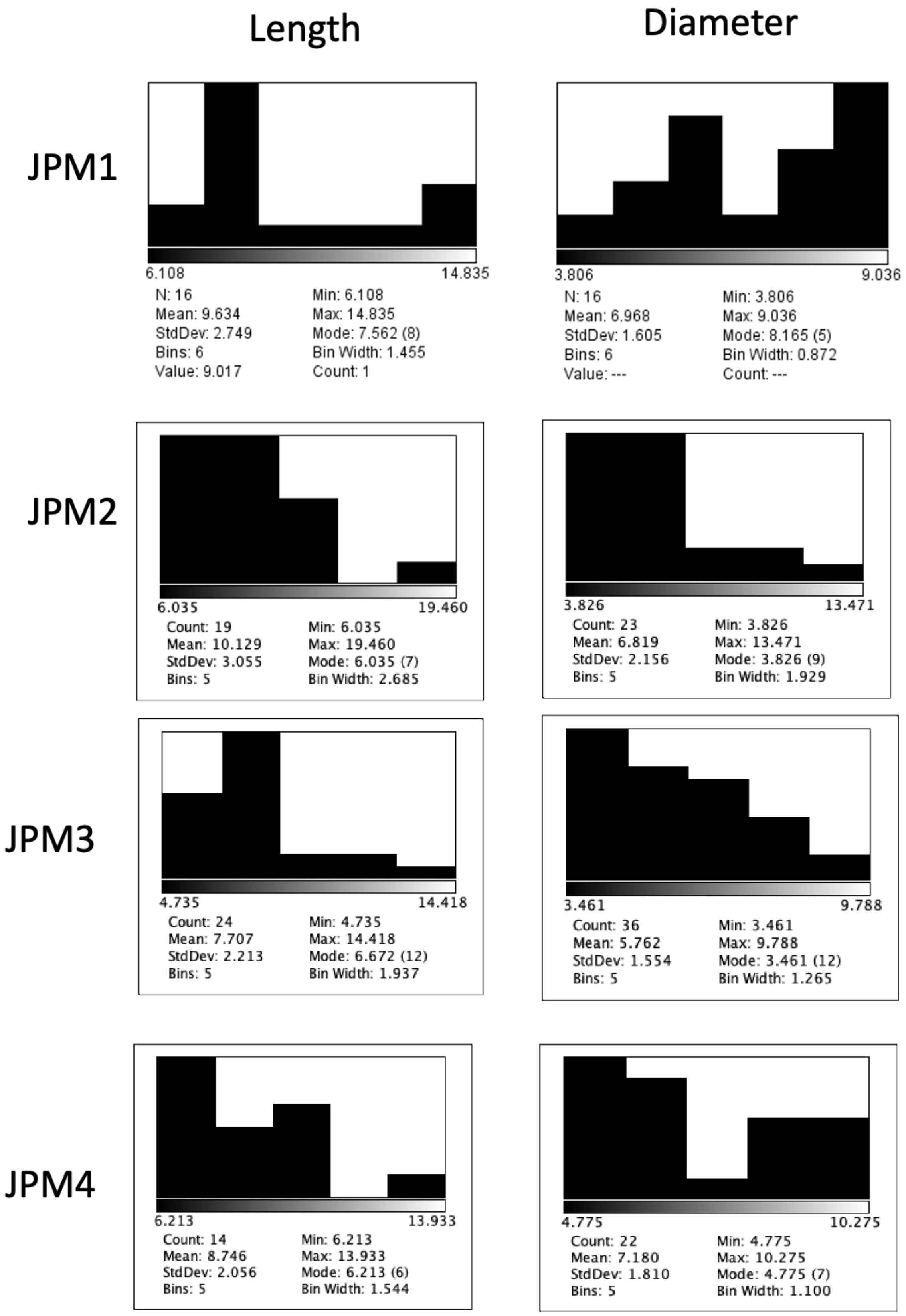

Figure S7. Distribution of diameter and length of JPM1, JPM2, JPM3 and JPM4

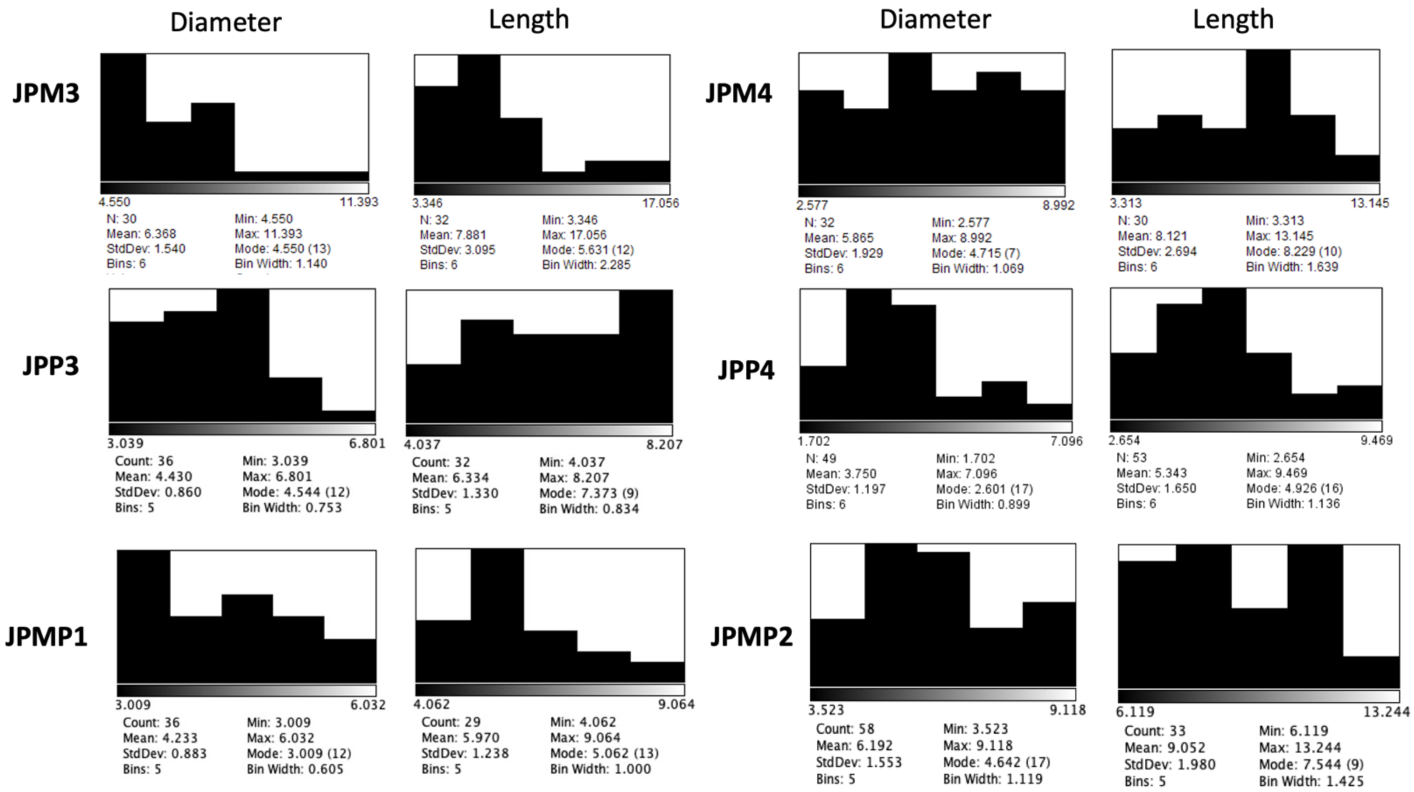

Figure S8. Distributions of diameter and length of all particle types analyzed by SEM

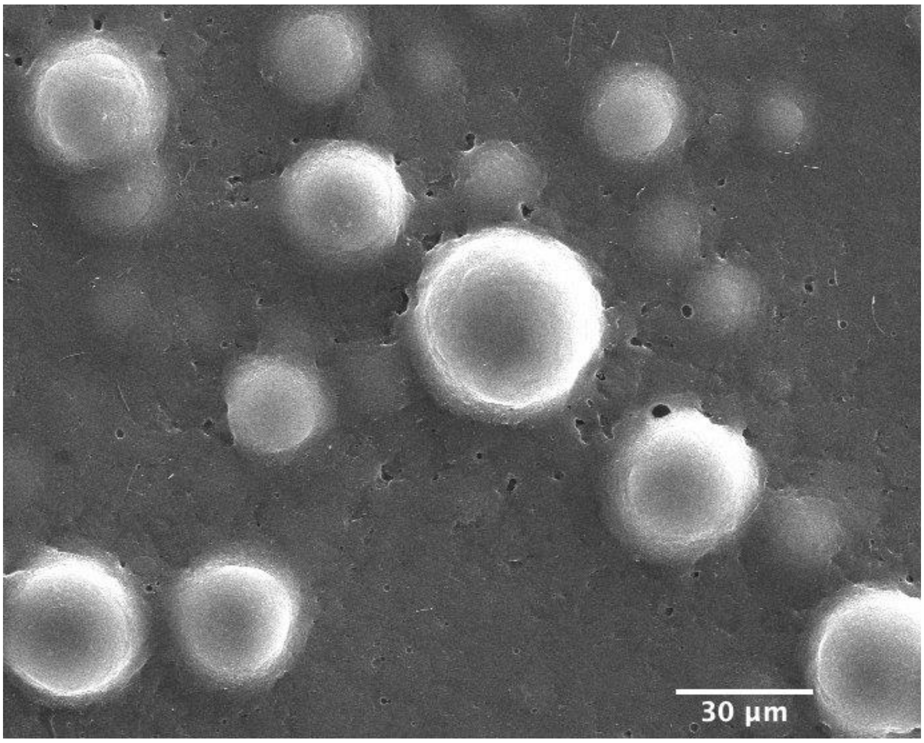

Figure S9. SEM images of PCL particles

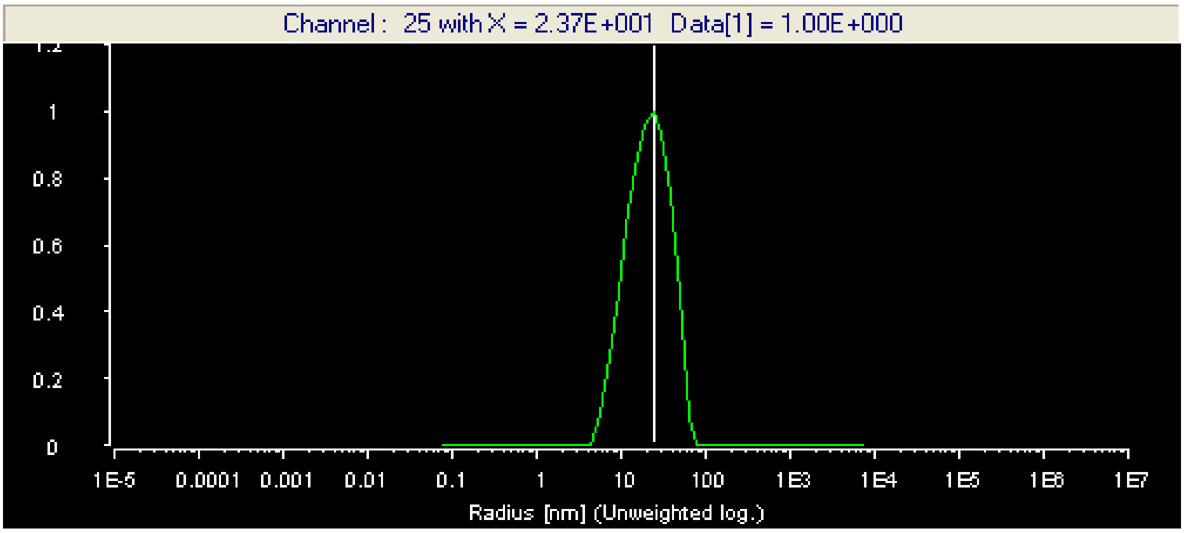

Figure S10. The hydrodynamic radius of PPYNP

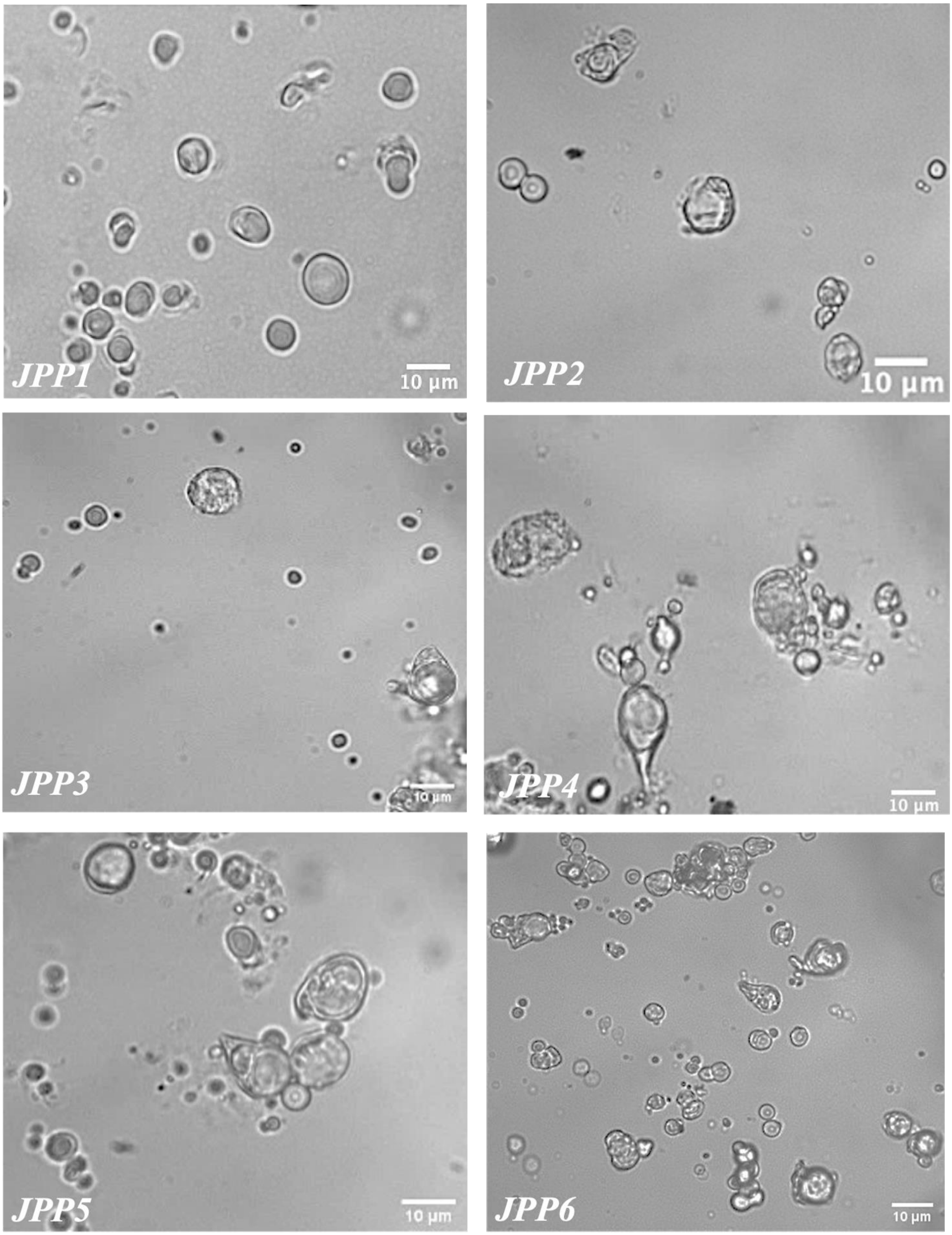

Figure S11. Light microscope images of JPPs prepared by using different ratios of PPYNP

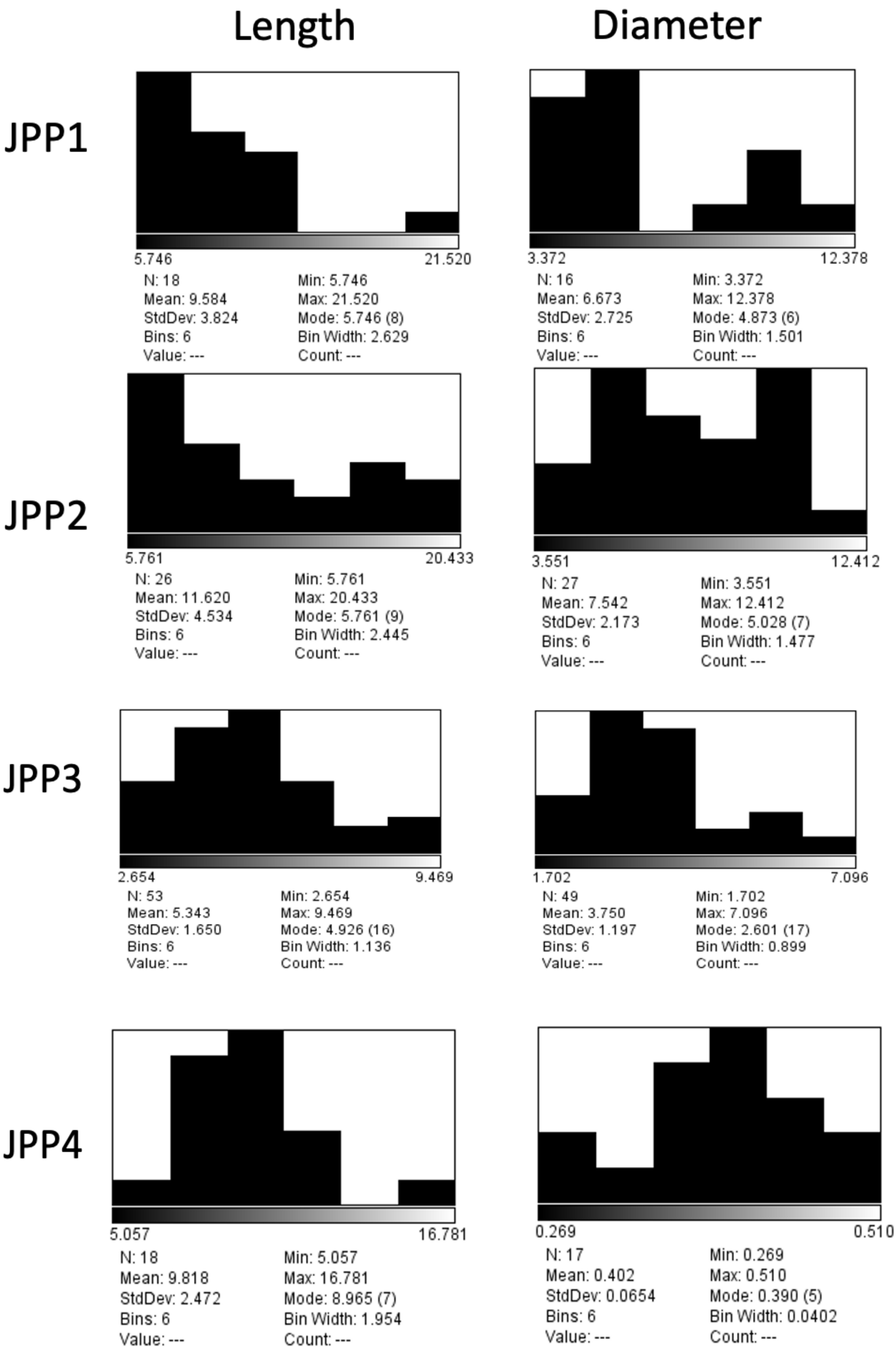
Length
Diameter
JPP1
5.746
21.520
N: 18
Mean: 9.584
StdDev: 3.824
Bins: 6
Value: ---
Min: 5.746
Max: 21.520
Mode: 5.746 (8)
Bin Width: 2.629
Count: ---
3.372
12.378
N: 16
Mean: 6.673
StdDev: 2.725
Bins: 6
Value: ---
Min: 3.372
Max: 12.378
Mode: 4.873 (6)
Bin Width: 1.501
Count: ---
JPP2
5.761
20.433
N: 26
Mean: 11.620
StdDev: 4.534
Bins: 6
Value: ---
Min: 5.761
Max: 20.433
Mode: 5.761 (9)
Bin Width: 2.445
Count: ---
3.551
12.412
N: 27
Mean: 7.542
StdDev: 2.173
Bins: 6
Value: ---
Min: 3.551
Max: 12.412
Mode: 5.028 (7)
Bin Width: 1.477
Count: ---
JPP3
2.654
9.469
N: 53
Mean: 5.343
StdDev: 1.650
Bins: 6
Value: ---
Min: 2.654
Max: 9.469
Mode: 4.926 (16)
Bin Width: 1.136
Count: ---
1.702
7.096
N: 49
Mean: 3.750
StdDev: 1.197
Bins: 6
Value: ---
Min: 1.702
Max: 7.096
Mode: 2.601 (17)
Bin Width: 0.899
Count: ---
JPP4
5.057
16.781
N: 18
Mean: 9.818
StdDev: 2.472
Bins: 6
Value: ---
Min: 5.057
Max: 16.781
Mode: 8.965 (7)
Bin Width: 1.954
Count: ---
0.269
0.510
N: 17
Mean: 0.402
StdDev: 0.0654
Bins: 6
Value: ---
Min: 0.269
Max: 0.510
Mode: 0.390 (5)
Bin Width: 0.0402
Count: ---

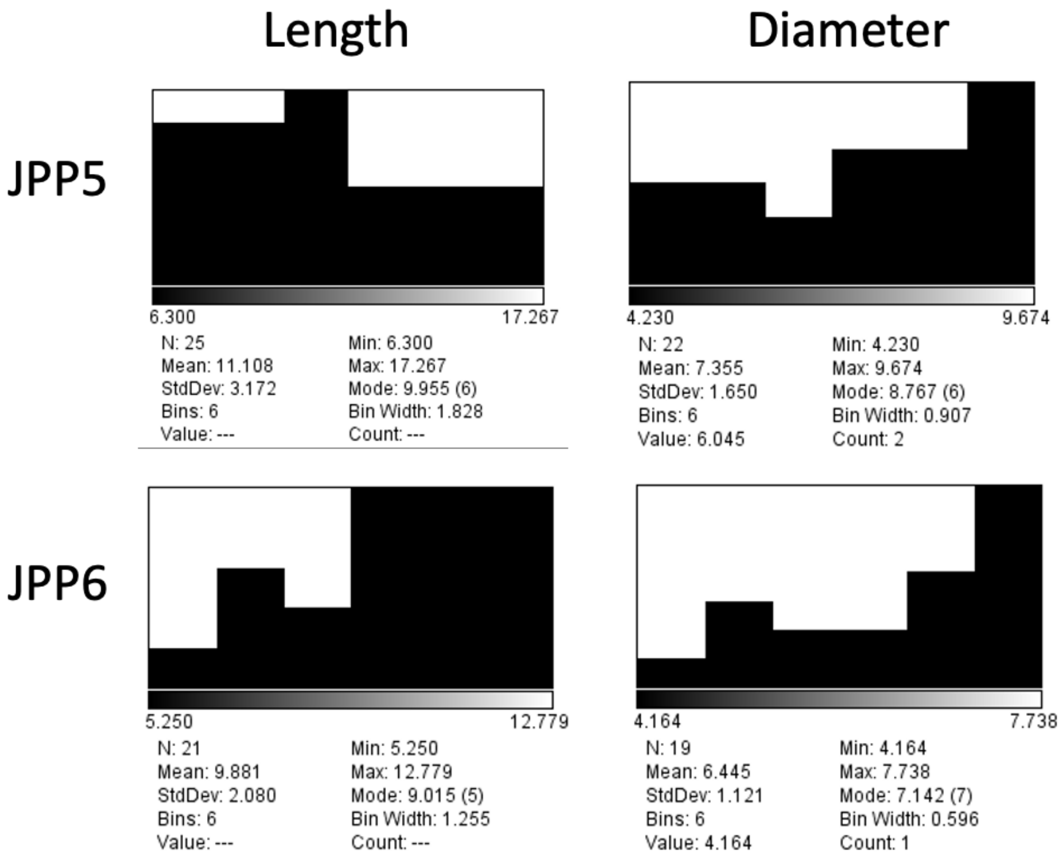

Figure S12. Distribution of diameter and length of JPP1, JPP2 JPP3, JPP4, JPP5 and JPP6

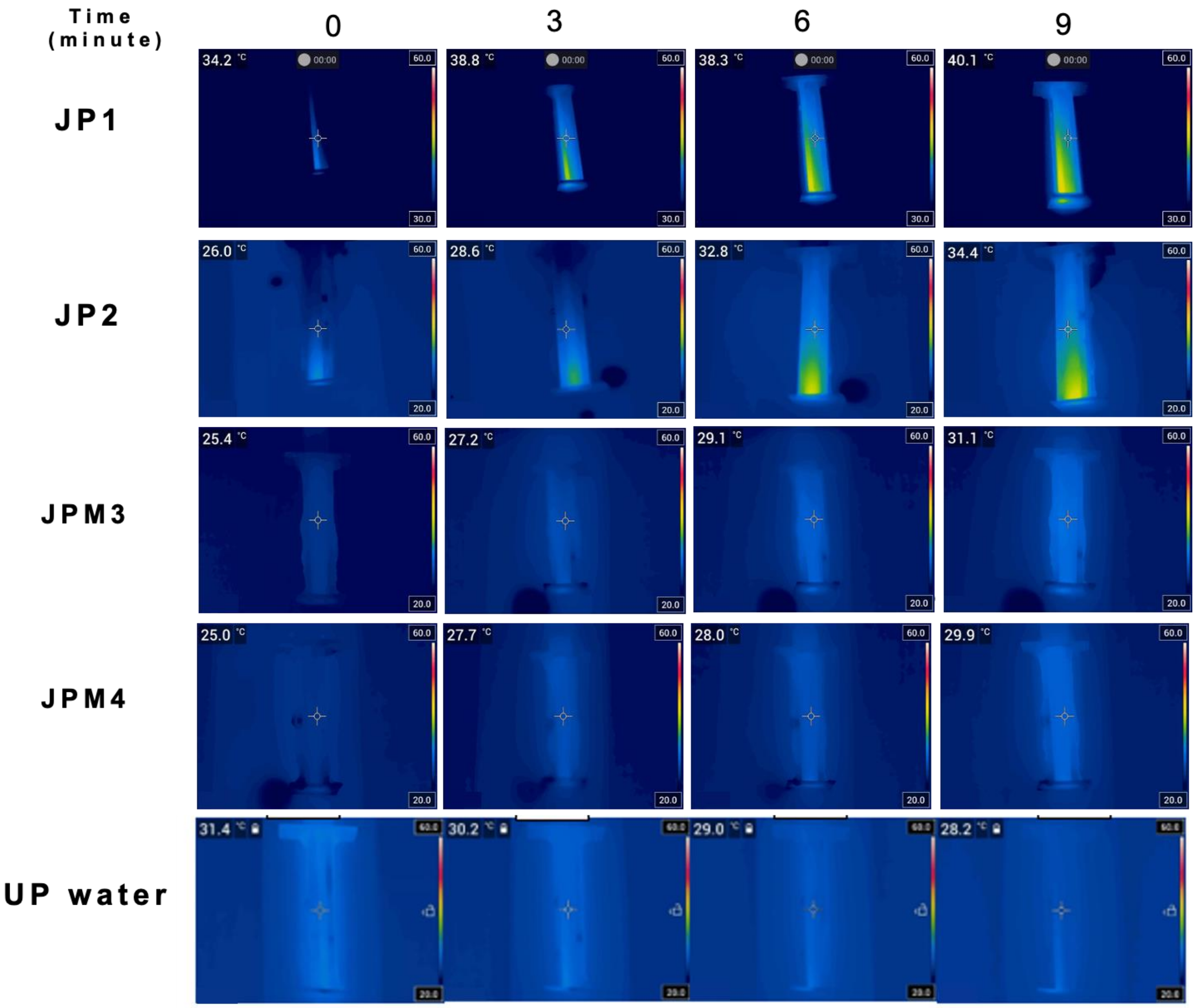

Figure S13. Thermal camera images of JP1, JP2, JPM3, and JPM4 containing different surfactants and UP water to observe temperature change in UP water at 808 nm NIR irradiation and 2.5W power

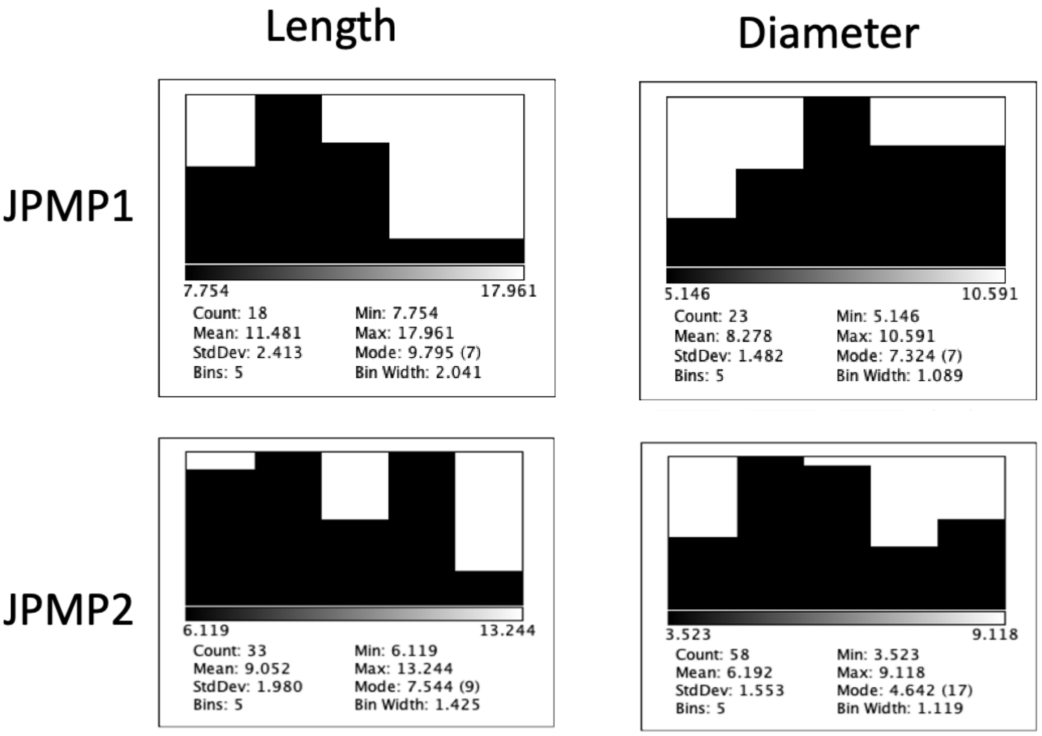

Figure S14. Distribution of diameter and length of JPMP1 and JPMP2.

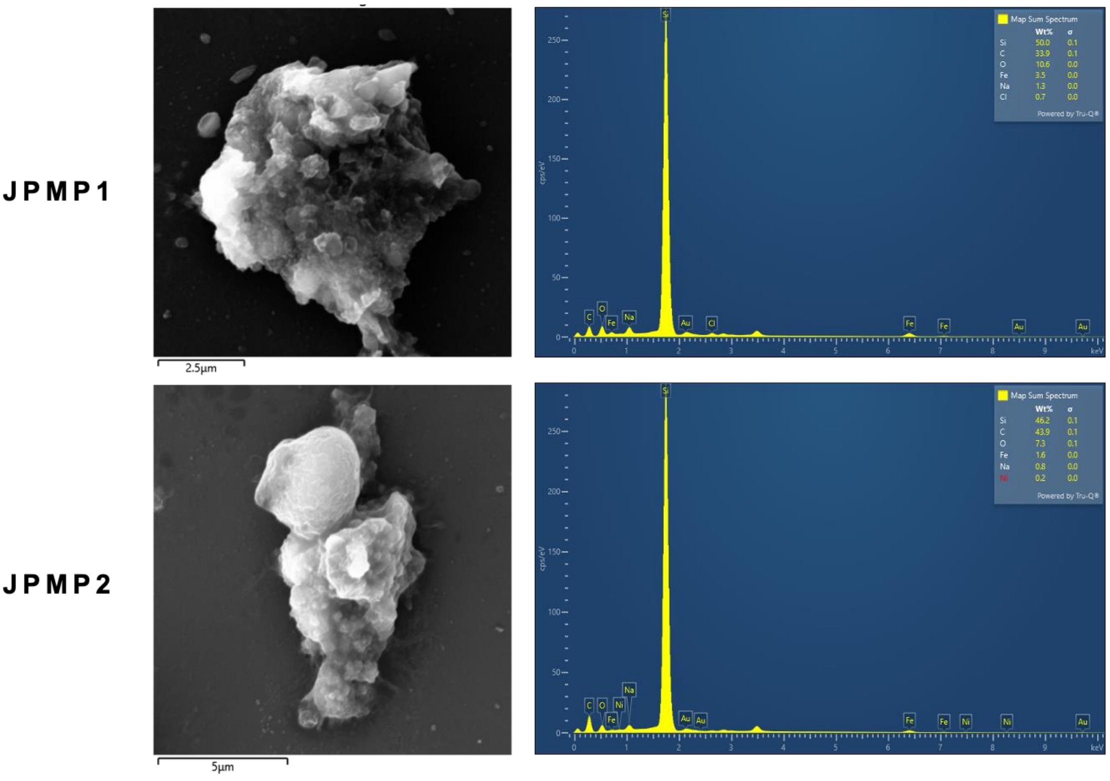

Figure S15. EDX Analysis of JPMP1 with x1 PPYNP and x2 MNPs (external phase containing PVA/SDBS co-surfactants) JPMP2 with x1 PPYNP and x2 MNPs (external phase containing SDS/SDBS co-surfactants)

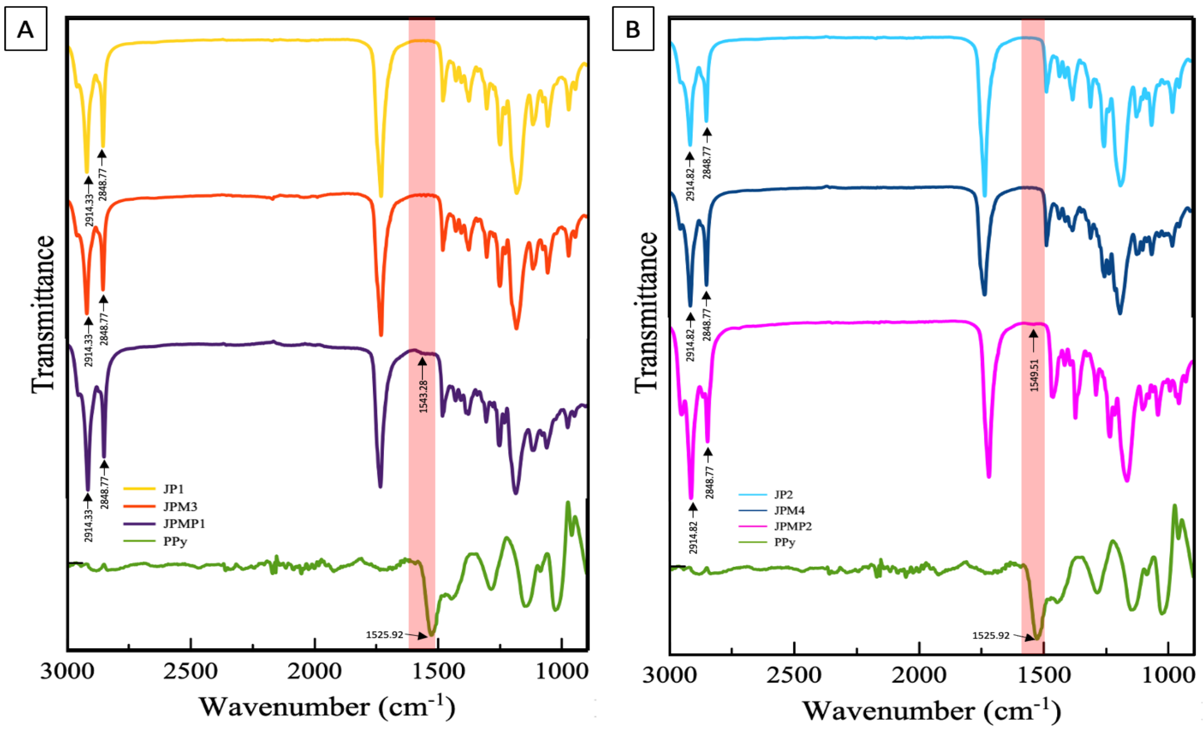

Figure S16. (a) Fourier transform infrared (FTIR) spectra of A) JP1, JPM3, JPMP1, and PPYNP and B) JP2, JPM4, JPMP2, and PPYNP. JP1 and JP2 were prepared with PVA/SDBS and SDS/SDBS co-surfactants. JPM3 and JPM4: external phase containing PVA/SDBS and SDS/SDBS co-surfactants, respectively (x2 MNPs). JPMP1 with x1 PPYNP and x2 MNPs (external phase containing PVA/SDBS co-surfactants) JPMP2 with x1 PPYNP and x2 MNPs (external phase containing SDS/SDBS co-surfactants)

Photothermal conversion efficiency (η) is calculated according to the following equation [3];

$$\eta = \frac{hS(T_{max}-T_{surr})-Q_{dis}}{I(1-10^{-\lambda})}$$ ……………………………..Eq. S2

η; photothermal conversion efficiency

h: heat transfer coefficient

S: surface area of the quartz bat

Tmax : maximum steady temperature of the dispersion

Tsurr : environmental temperature

Qdis : the heat emitted from the absorbed light with both the quartz cuvette cell and the dispersion

I : laser power (2W)

λ; absorbance value of JPs at 808 nm wavelength (1.042)

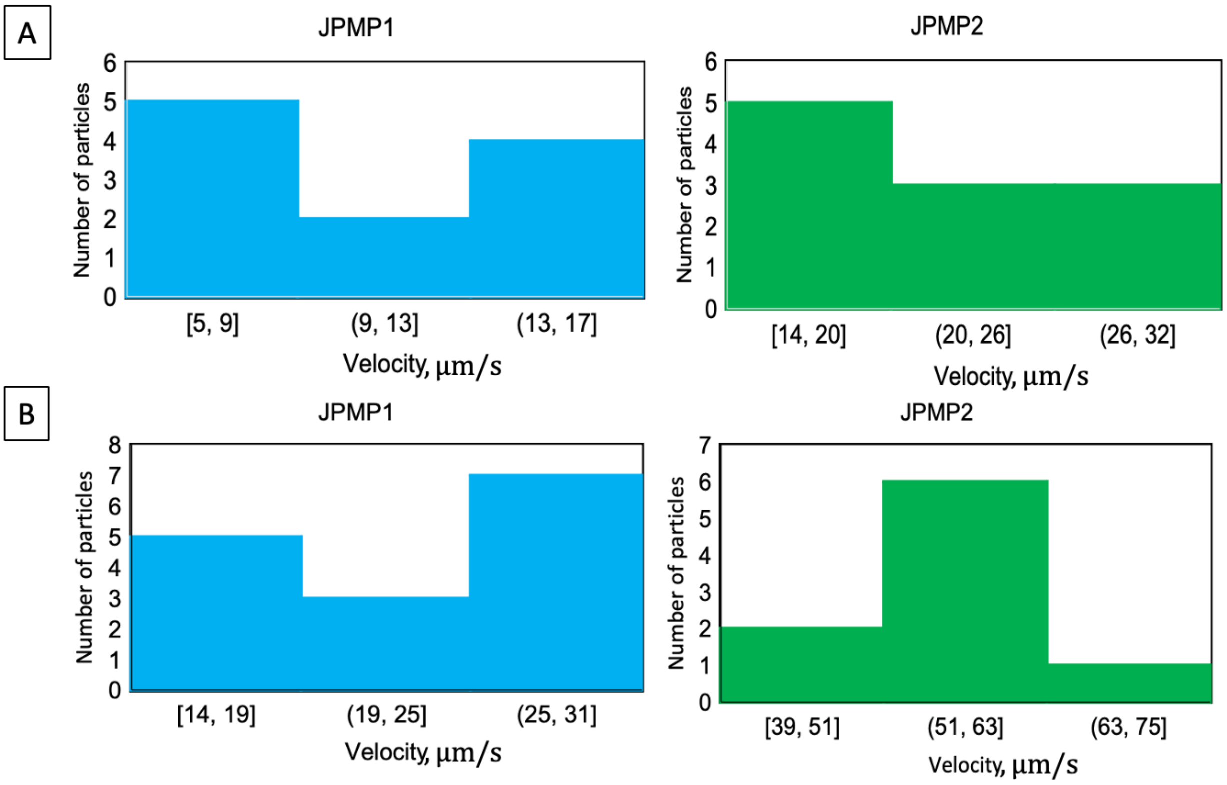

Figure S17. Distribution of velocity of A) magnetic and B) NIR-driven JPMP1 and JPMP2.

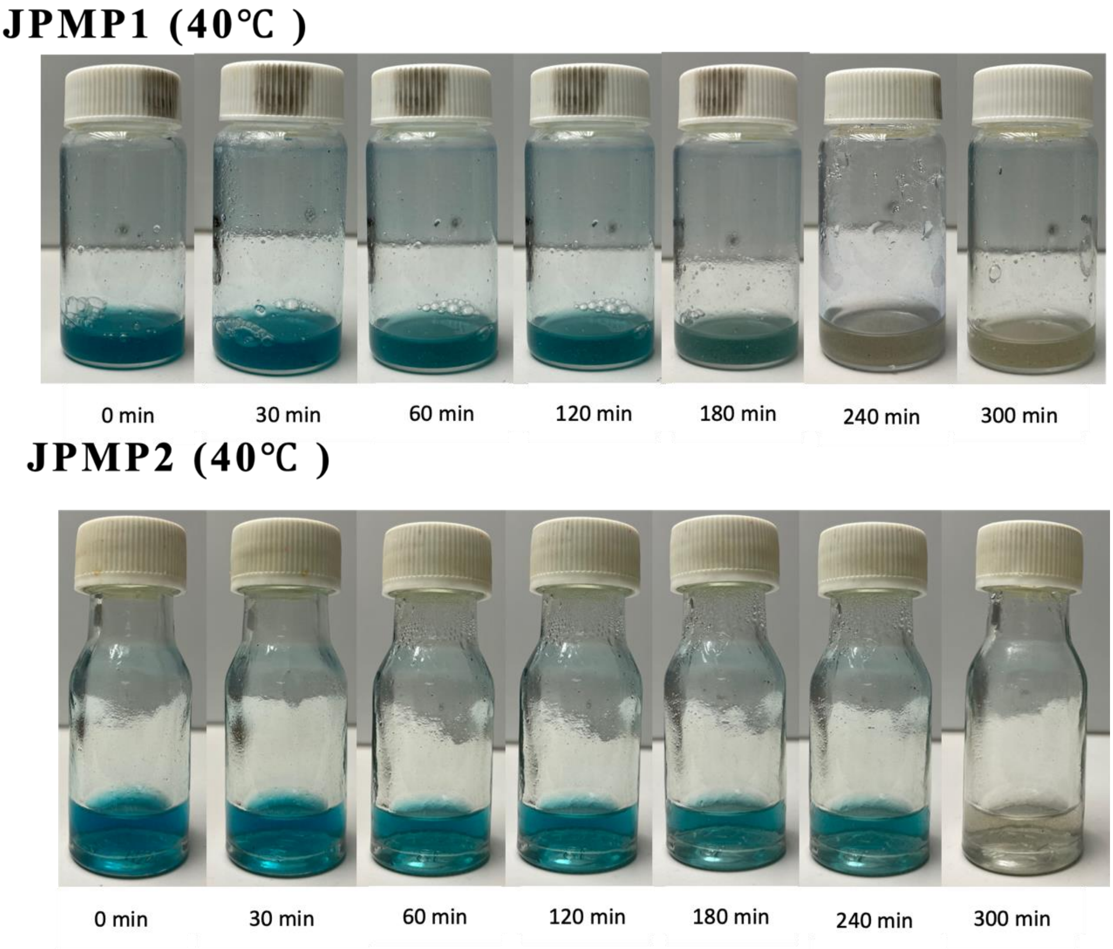

Figure S18. Decolorization of MB as a function at a fixed reaction time of 300 min in Fenton reactions with JMs at 40°C

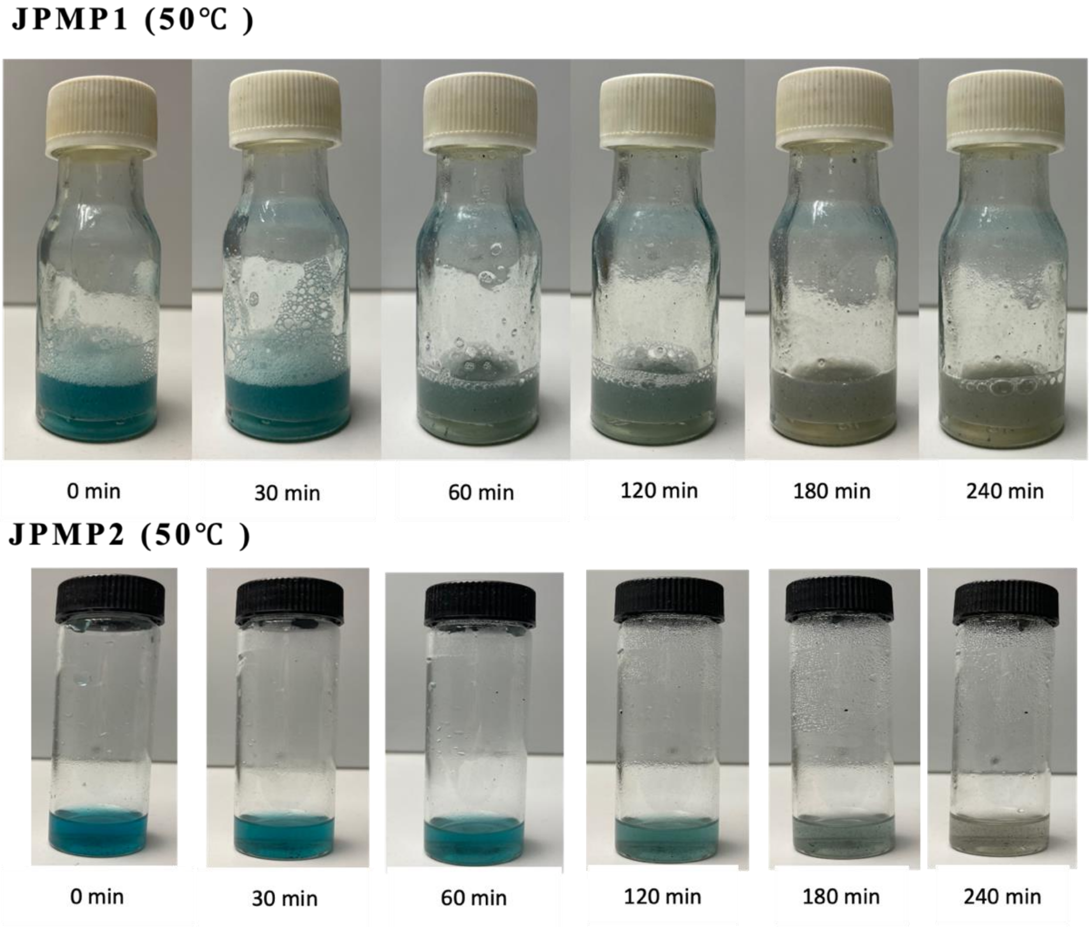

Figure S19. Decolorization of MB as a function at a fixed reaction time of 300 min in Fenton reactions with JMs at 50^oC

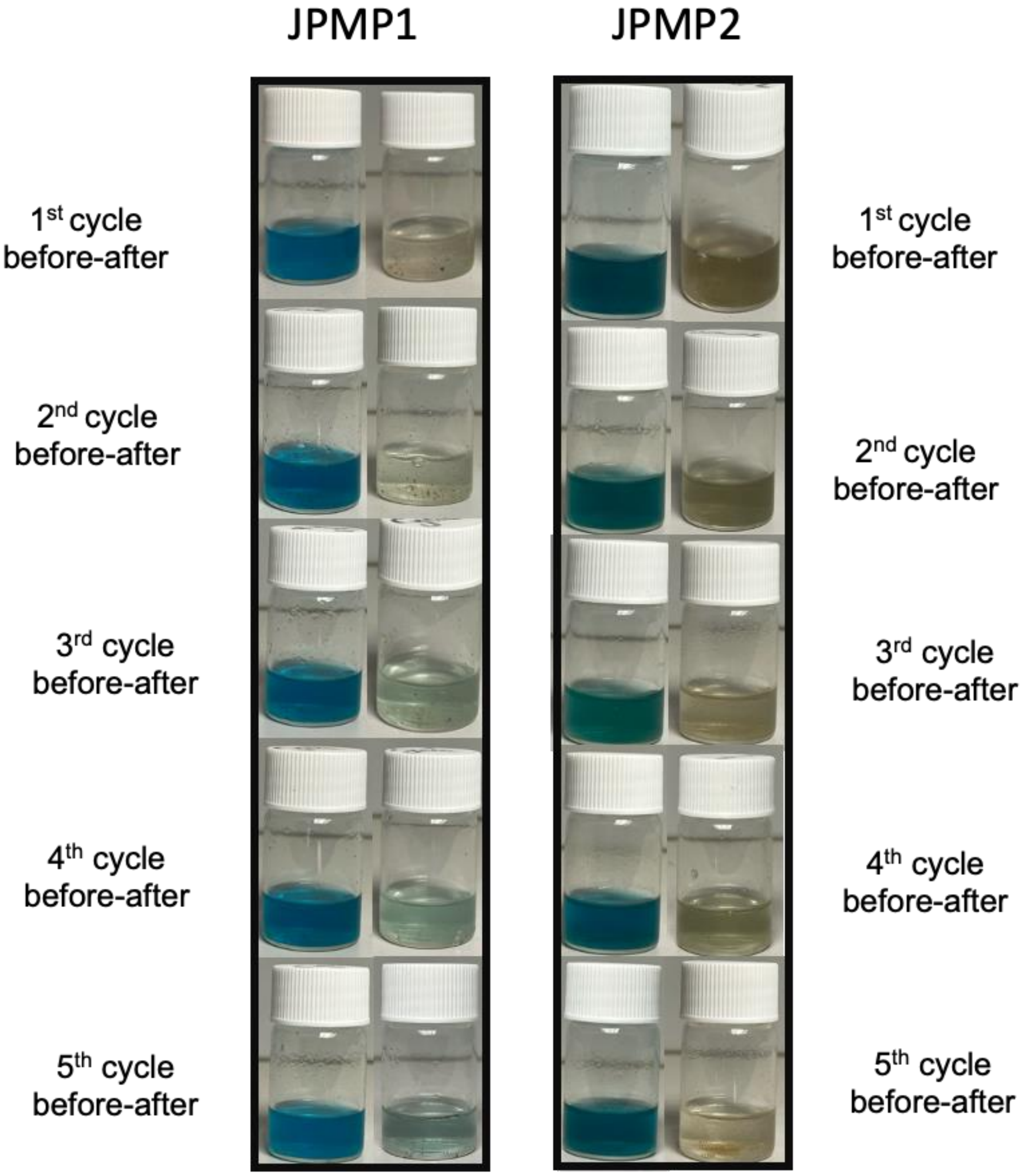

Figure S20. Visual inspection of the color change of the MB solution before and after catalytic Fenton reaction conducted at 40°C

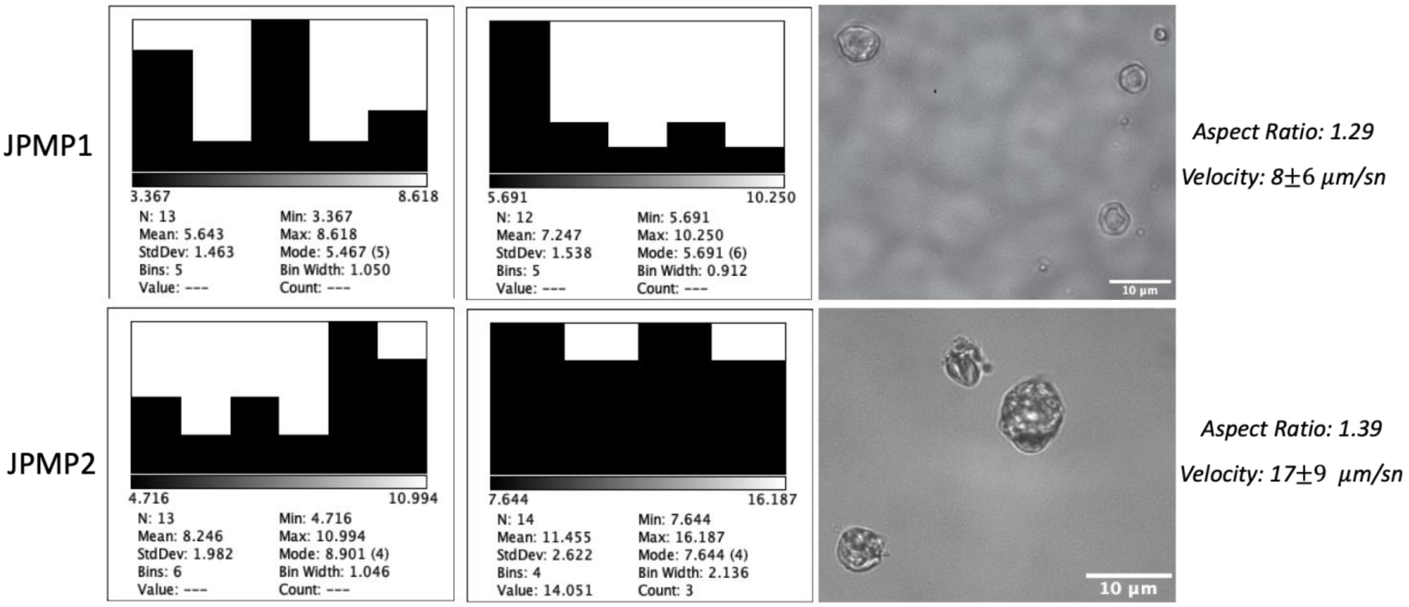

Figure S21. Characterization of the size of the JPMP1 and JPMP2 micromotors at the end of 5 the cycles of Fenton reaction and corresponding velocity of Janus microrobots upon application of magnetic field (480-mT).

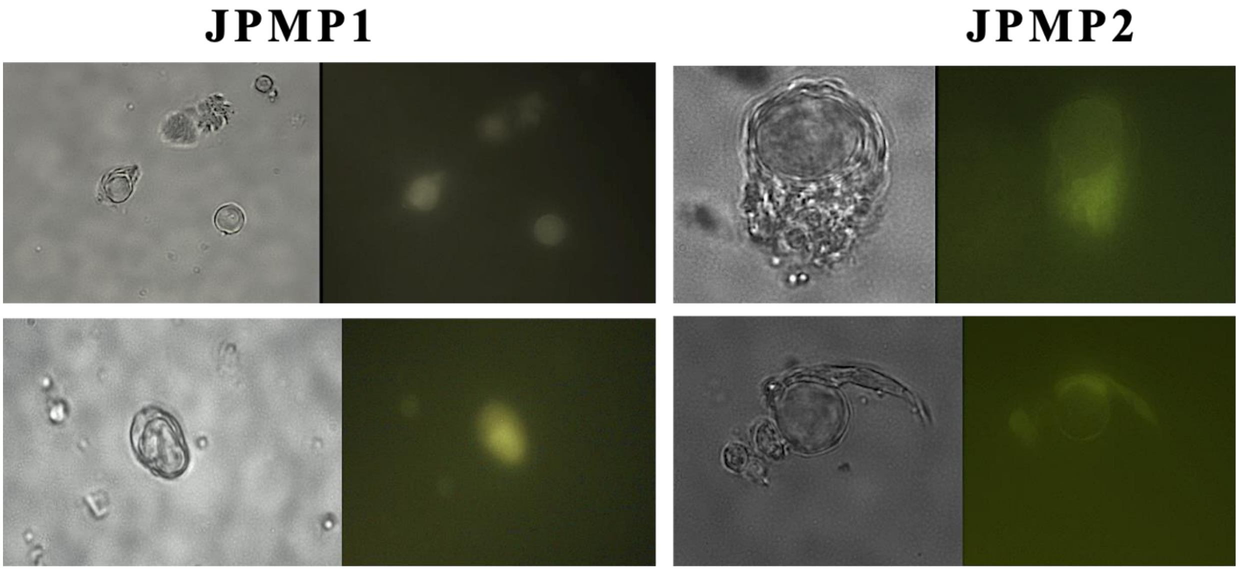

Figure S22. Optical microscope and fluorescence microscope images of JPMP1 and JPMP2 containing Rhodamin B 24 hours after phase separation

References

(1) Fan, Y. L.; Tan, C. H.; Lui, Y.; Zudhistira, D.; Loo, S. C. J., Mechanistic Formation of Drug-Encapsulated Janus Particles Through Emulsion Solvent Evaporation. *RSC Advances* **2018,** *8* (29), 16032-16042.

(2) van Zyl, A. J. P.; Sanderson, R. D.; de Wet-Roos, D.; Klumperman, B., Core/Shell Particles Containing Liquid Cores: Morphology Prediction, Synthesis, and Characterization. *Macromolecules* **2003,** *36* (23), 8621-8629.

(3) Liu, X.; Li, B.; Fu, F.; Xu, K.; Zou, R.; Wang, Q.; Zhang, B.; Chen, Z.; Hu, J., Facile synthesis of Biocompatible Cysteine-coated CuS Nanoparticles with High Photothermal Conversion Efficiency for Cancer Therapy. *Dalton Transactions* **2014,** *43* (30), 11709-11715.
